# Accuracy of Navigation and Robot-Assisted Systems for Dental Implant Placement: A Systematic Review

**DOI:** 10.3390/dj13110537

**Published:** 2025-11-14

**Authors:** Daria Pisla, Vasile Bulbucan, Mihaela Hedesiu, Calin Vaida, Alexandru Pusca, Rares Mocan, Paul Tucan, Cristian Dinu, Doina Pisla

**Affiliations:** 1 Department of Maxillofacial Surgery and Radiology, Oral Radiology, “Iuliu Hatieganu” University of Medicine and Pharmacy, 32 Clinicilor Street, 400006 Cluj-Napoca, Romania; daria.pisla@elearn.umfcluj.ro (D.P.); mhedesiu@umfcluj.ro (M.H.); 2 CESTER, (Research Center for Industrial Robots Simulation and Testing), Department of Mechanical Systems Engineering, Faculty of Industrial Engineering, Robotics and Production Management Technical University of Cluj-Napoca, 28 Memorandumului Street, 4000114 Cluj-Napoca, Romania; vasile.bulbucan@mep.utcluj.ro (V.B.); alexandru.pusca@mep.utcluj.ro (A.P.); paul.tucan@mep.utcluj.ro (P.T.); doina.pisla@mep.utcluj.ro (D.P.); 3 Department of Maxillofacial Surgery and Radiology, Maxillofacial Surgery and Implantology, “Iuliu Hatieganu” University of Medicine and Pharmacy, 37 Iuliu Hossu Street, 400429 Cluj-Napoca, Romania; cristian.dinu@umfcluj.ro

**Keywords:** oral surgery, dental implant, navigation system, robot-assisted oral surgery, dental implant accuracy, systematic review

## Abstract

**Background**: Computer-assisted implant surgery (CAIS) aims to improve placement accuracy versus freehand drilling. We compared the three-dimensional accuracy of robot-guided CAIS (r-CAIS), dynamic navigation (d-CAIS), static-template guidance (s-CAIS), and freehand (FH) in clinical and in vitro settings. **Methods**: We searched PubMed/MEDLINE, Scopus, and Web of Science (1 January 2019–2025). Eligible populations were adults receiving conventional or zygomatic implants in vivo, plus validated in vitro human-jaw models using plan-versus-placement workflows; studies had to report study-level means with dispersion for ≥1 primary outcome with ≥5 implants per arm. Interventions were r-CAIS, d-CAIS, or s-CAIS; with a baseline as the freehand technique. Risk of bias used RoB 2 (RCTs), ROBINS-I (non-randomized clinical), and QUIN (in vitro). Because of heterogeneity in definitions and workflows, we performed a descriptive synthesis by modality (no meta-analysis). **Registration**: OSF. **Results**: Forty-three studies (7 RCTs, 10 non-randomized clinical, 26 in vitro) reported more than 4000 implants. Across studies, typical study-level means for global linear deviation clustered around < 1 mm (r-CAIS), ~1 mm (d-CAIS), ~1.3 mm (s-CAIS), and ~1.8 mm (FH). In clinical contexts, d-CAIS often showed slightly lower angular deviation than s-CAIS. **Conclusions**: CAIS improves accuracy versus freehand. d-CAIS and s-CAIS show similar linear accuracy, with d-CAIS frequently yielding slightly lower angular deviation; r-CAIS exhibits tight error clusters in our dataset, but limited comparative clinical evidence precludes superiority claims. **Limitations**: non-uniform registration/measurement, variable operator experience, and absence of meta-analysis.

## 1. Introduction

Dental implants have become a routine solution for edentulism, with high long-term success rates reported in clinical studies [1]. However, the precision of implant positioning is critically important for the success of the final prosthesis. Implants placed at an improper angulation or depth can complicate prosthetic rehabilitation, impair bone integration, develop facial asymmetry due to an incorrect occlusion and even risk damaging vital anatomical structures such as nerves or the maxillary sinus [2,3]. Consequently, achieving a prosthetically driven, accurate implant placement is widely regarded as a key factor in avoiding early or late implant failures [2,3]. As a conclusion, surgical accuracy is a critical factor for both functional and aesthetic outcomes, motivating its detailed analysis across multiple technological support platforms and freehand interventions.

Over the past two decades, clinicians have increasingly turned to technology to increase placement accuracy and overcome the limitations of freehand surgery. Traditional freehand implantation relies only on the surgeon’s skill and experience, which can lead to positional deviations; computer-assisted implant surgery (CAIS) techniques were introduced, evolving through static, dynamic, and now robotic guidance systems [1]. In static guided surgery, a custom surgical template (guide) is fabricated from pre-operative cone-beam CT data and digital planning to direct the drill in a fixed trajectory, reproducing the planned implant position [1]. The term “static guide” does not denote a single standardized surgical technique but rather encompasses a range of methods that share the principle of preoperative, computer-assisted planning for implant placement. Static guides vary in support type (tooth-, mucosa-, bone-, or pin-supported), extent of guidance (pilot-only or fully guided), and fabrication method (conventional or digitally designed and 3D-printed). Design features such as sleeve geometry, preset offsets, bur–sleeve tolerances, manufacturing accuracy, and guide stabilization further influence both linear and angular deviations. These variations can significantly affect surgical accuracy and therefore confound direct comparisons with dynamic navigation (d-CAIS) and robot-assisted systems (r-CAIS) [4]. In contrast, dynamic navigation involves real-time tracking of the handpiece, allowing the surgeon to actively control drilling while visualizing the planned versus actual trajectory. Unlike static guidance, no mechanical constraint is applied during drilling, providing greater intraoperative flexibility at the cost of a steeper learning curve in interpreting navigation feedback. Various systems such as fully autonomous dental implant robots have been prototyped, as seen with systems like China’s Remebot (Beijing Bihui Weikang Technology Co., Ltd., Beijing, China), which can carry out the osteotomy and implant insertion according to the virtual plan [1]. These advancements have a clear trend in implantology from freehand techniques toward increasing computerization and mechanization with the aim of greater accuracy and consistency in outcomes.

Recent high-quality meta-analyses, including those by Khan et al., 2024 [5], and Khaohoen et al., 2024 [1] have provided compelling evidence for a relative accuracy hierarchy among static, dynamic, and robotic computer-assisted implant surgery (CAIS) techniques. Static and dynamic guidance systems consistently demonstrate higher positional and angular accuracy compared with conventional freehand placement, while robotic platforms show further improvements in precision and reproducibility. Despite these advances, substantial heterogeneity remains across study designs, outcome definitions, and operator-related factors, and several clinically relevant subgroups continue to be underrepresented in pooled analyses.

Accordingly, this systematic review was designed to extend previous benchmark meta-analyses and synthesize evidence published between 2019 and 2025. Its specific aims are to standardize outcome definitions across heterogeneous clinical, in vitro, and in vivo workflows to enable valid inter-modality comparisons of accuracy. Integrate underrepresented subgroups, including hybrid static–dynamic protocols and zygomatic implant procedures, which have been inconsistently included in earlier reviews. Evaluate the influence of operator learning and training, parameters largely omitted from prior syntheses, on surgical accuracy and reproducibility. Delineate the current technological frontier of CAIS by identifying the limited number of cadaveric and artificial intelligence–assisted validation studies available to date.

By addressing these objectives, this review serves as an analytical extension of prior meta-analyses, contextualizing their conclusions with the most recent evidence and providing a unified assessment of accuracy trends, learning-curve dynamics, and technological evolution across static (s-CAIS), dynamic (d-CAIS), and robotic (r-CAIS) implant surgery systems.

## 2. Methods

### 2.1. Protocol and Registration

This systematic review was registered on the Open Science Framework (OSF; https://doi.org/10.17605/OSF.IO/98Q3G, accessed on 30 July 2025). Although our review protocol was registered on OSF after data collection was finalized, all eligibility criteria and analysis strategies were pre-specified and adhered to throughout the study.

### 2.2. Study Question and PICOS Strategy

The review was framed according to the PICOS model. Population (P) comprised partially or fully edentulous human patients, cadavers, as well as validated human-jaw replicas fabricated from polyurethane or 3-D printing. Interventions (I) included any r-CAIS, d-CAIS, or s-CAIS workflow executed with commercially available or prototype systems, optical navigation units (e.g., Navident—ClaroNav Technology Inc., Toronto, ON, Canada^®^), and stereolithographic drill guides (e.g., coDiagnostiX—Dental Wings Inc., Montreal, QC, Canada). Comparators (C) were either freehand placement or an alternative CAIS modality tested under the same conditions. Conventional root-form implants, and zygomatic implants were present in the dataset. Outcomes (O) were primary accuracy metrics; global three-dimensional linear deviation at the platform and apex, vertical (depth) deviation, and angular deviation between the planned and achieved implant axes; secondary outcomes included operative time, registration or guide-fabrication time, intra-operative complications and the need for corrective maneuvers. Study designs (S) eligible for inclusion were randomized or non-randomized clinical trials, prospective or retrospective cohort studies, and controlled laboratory investigations that analyzed at least five implants per study arm.

The review question was therefore: In human jaws or validated jaw models, do robot-assisted and other computer-assisted implant surgery (CAIS) techniques achieve greater three-dimensional positional accuracy than conventional freehand placement, and how does accuracy differ among the three CAIS modalities (static, dynamic, and robotic)?

### 2.3. Eligibility Criteria

English-language articles were considered when they (i) described clinical or in vitro drilling of dental implants, (ii) applied at least two CAIS modality of interest, and (iii) quantified accuracy by superimposing post-operative cone-beam CT, optical surface scan, or coordinate-measurement data on the virtual plan and reporting mean ± standard deviation or raw values. Exclusion criteria included narrative or systematic reviews, technical notes, conference abstracts, editorials, animal studies, finite-element simulations, reports with fewer than five implants per group, studies with no comparative arm, mini-implants and papers that evaluated only prosthetic fit or crestal bone levels without measuring positional deviation.

### 2.4. Study-Selection and Data-Extraction Process

Each included report was coded for publication year, study design (randomized clinical trial, non-randomized clinical cohort, or controlled in vitro experiment), guidance modality, number of implants analyzed, registration method, outcome definitions and numerical accuracy data. Data was extracted independently and imported into Rayyan [6] by the two reviewers (V.B, P.T). When a paper provided both platform-to-plan and apex-to-plan deviations, both were captured; when accuracy was expressed as root-mean-square or absolute means, values were converted to pooled means and standard deviations. Disagreements in either the selection or extraction phase were resolved by discussion and, where necessary, by referring to a third investigator (C.V). The complete flow of records is documented in a PRISMA 2020 diagram [7], and a PRISMA Checklist was provided as Supplementary Table S1.

### 2.5. Search Strategy

A comprehensive electronic search was conducted by V.B and D.P, with oversight from C.V and consultation with a colleague experienced in systematic reviews (M.H) in the three databases; PubMed/MEDLINE, Scopus and Web of Science Core Collection from 2019 to 2025, with English-only publications and no restrictions on publication type applied. PubMed was selected for its exhaustive coverage of dental-implant literature; Scopus and Web of Science were added to capture records indexed outside MEDLINE and to enable transparent deduplication. The three-database search corresponds with the recommendation of PRISMA 2020 Guidelines [7] and Cochrane Handbook guidance [8]. The core syntax combining MeSH headings and free-text terms is shown in Table 1:

The Scopus search was deliberately restricted to the Dentistry subject area and to publications from 2019 to 2025 to align with the period in which contemporary navigation and robotic systems became commercially available; no language limits were applied in PubMed or Web of Science, although only the English language papers were selected. Grey-literature sources were not searched because positional-accuracy studies require postoperative imaging and are almost exclusively published in peer-reviewed journals.

Search strings were adapted to the indexing rules of the database. Titles and abstracts were screened independently by two reviewers (V.B, P.T); potentially eligible reports underwent full-text assessment, again in duplicate, with disagreements resolved by discussion. Duplicates were removed, and the selection process was summarized in a PRISMA 2020 flow diagram. Data extraction followed a spreadsheet completed independently by two reviewers (V.B, P.T); any discrepancy in retrieved record counts or search-string translation was discussed between V.B and P.T, unresolved items were adjudicated by a third reviewer (C.V), and any other issues were discussed with experienced personnel (M.H, D.P) in systematic reviews. If essential numerical values were missing, corresponding authors were contacted by e-mail.

### 2.6. Types of Outcome Measures

The review extracted three established accuracy variables.

➢ Coronal (platform) deviation is defined as the three-dimensional Euclidean distance, in millimetres, between the centre of the implant shoulder in the virtual plan and in the registered post-operative scan.
➢ Apical deviation is defined as a 3-D Euclidean distance (mm) between the centroid of the apical endpoint in the plan and in the registered post-operative model, defined analogously to the platform to ensure symmetry of reference points.
➢ Angular deviation represented the absolute inter-axis angle, in degrees, obtained from the arccosine of the dot-product of the planned and achieved implant vectors.

These definitions follow the CBCT super-imposition protocols described by Tao et al., 2022 [9] and Taheri Otaghsara et al., 2023 [10] for in vitro work and by Jorba-García et al., 2023 [11] for a split-mouth clinical trial and are identical to the method used in the multi-workflow bench comparison of Xu et al., 2024 [3]. Where authors reported separate Bucco-lingual and Mesio-distal offsets the vector norm was computed to yield a single global value, and when both “planned-versus-placed” and “placed-versus-ideal” data were available the former were extracted because they most directly reflect surgical trueness rather than prosthetic fit.

### 2.7. Risk-of-Bias Assessment

Risk of bias was assessed with design-specific, validated instruments.

➢ Randomized controlled trials (RCTs, n = 7) were appraised with the Cochrane RoB 2 tool (five domains).
➢ Non-randomized clinical investigations, including prospective or retrospective cohorts and case-series (n = 10), were assessed with ROBINS-I (seven domains).
➢ In vitro accuracy studies (n = 26) were analyzed with the QUIN checklist (five methodological domains).

Two reviewers worked independently after piloting each instrument on one representative article; disagreements were resolved by discussion between the two primary reviewers (V.B, P.T), and unresolved conflicts were resolved by a third reviewer (C.V). Domain-level judgements were entered into a spreadsheet that generated colour-coded overall ratings (“Low”, “Some concerns/Moderate”, “Serious/High”). Considering that no quantitative publication-bias assessment (e.g., Funnel plot) was undertaken because no meta-analysis was performed, the risk was judged within the relevant domain of each risk-of-bias tool. A ‘traffic-light’ figure and summary bar chart were created in Excel to visualize the findings.

### 2.8. Synthesis Methods

Given heterogeneity in design, setting (clinical, in vitro), implant category (conventional, zygomatic), and definitions, we performed a descriptive synthesis using grouped graphical displays by modality (r-CAIS, d-CAIS, s-CAIS, FH). Where subgroup data were separable in source studies, we summarize exploratory tendencies in text. No meta-analysis was undertaken.

We generated two complementary figure families. Main-text figures display all modalities on shared axes to enable between-modality comparisons. To enhance readability and allow within-modality inspection, we produced per-modality supplementary plots (Robot, Dynamic, Static, Freehand) for each outcome (Supplementary Figures S1–S12),showing study-level mean ± SD sorted by mean.

## 3. Results

### 3.1. Identification and Screening

**Figure 1:**
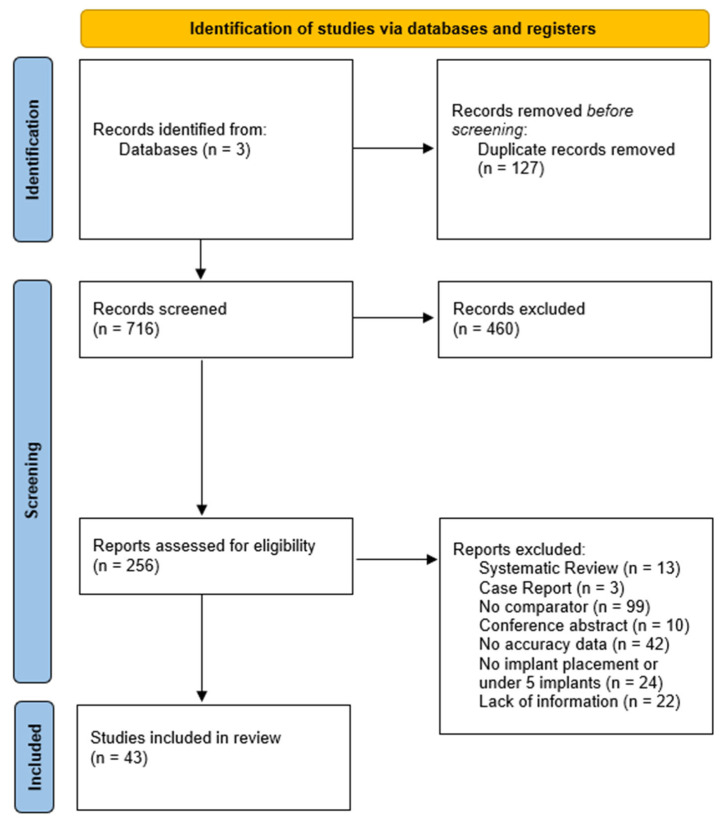
Preferred reporting items for systematic reviews and meta-analyses (PRISMA) flowchart summarizing the screening process.

The three-database search yielded 843 records (Figure 1, PRISMA 2020). After removal of 127 duplicates, 716 unique records were screened at title/abstract level, excluding 460. Two-hundred and fifty-six full-text articles were assessed for eligibility. Of these, 208 were excluded: 13 systematic reviews, 3 case reports, 99 non-comparative reports, 10 conference abstracts, 42 without quantitative accuracy data, 24 with no implant placement or <5 implants, and 22 lacking essential methodological information, including cadaveric investigation that did not meet the eligibility criteria. This left 43 primary investigations for qualitative synthesis.

### 3.2. Study Range and Characteristics

The forty-three primary studies encompassed a heterogeneous yet well-balanced mix of experimental designs. Seven were parallel or split-mouth randomized controlled trials (e.g., Kaewsiri et. al., 2019 [12]), and others were prospective or retrospective clinical cohorts. Clinical investigations analyzed an average of 28 implants per article (range 10–90), whereas laboratory studies drilled an average of 40 osteotomies (range 15–240). The final dataset spans publications from 2019 to 2025 and comprises approximately 4200 individual implants: 544 freehand, 753 static-guide, 2313 dynamic-navigation, and 595 robotic placements. Surgical sites were evenly distributed between maxillae and mandibles; 22% of cohorts involved fully edentulous arches, 55% partially edentulous jaws, and 23% single-tooth sites. The navigation technologies represented in the data were well represented across the dataset.

RCAIS was evaluated in 9 of the 43 primary studies, and most of these employed the fully autonomous Remebot system, which were in vitro usability experiments demonstrating Remebot’s consistent depth and angulation control [13,14,15,16]. Dynamic navigation (d-CAIS) featured in 36 studies: the majority used NaviDent as the navigation technology (e.g., [11,17,18]), some employed the Iris-100 platform, and two reported on custom-built optical prototypes [19,20]. Static computer-aided implant surgery (s-CAIS) was investigated in 24 papers, with coDiagnostiX^®^-designed stereolithographic guides predominating (eleven studies), and the remainder relying on BlueSkyPlan or generic STL-based workflows. Conventional freehand drilling served as a comparator in 21 investigations, notably the split-mouth RCT of Jorba-García et al., 2023 [11] and multiple in vitro bench studies underscoring the operator-dependent variability of unguided implant placement.

Software heterogeneity was pronounced: three studies combined mixed-reality or augmented-reality overlays with Navident^®^; two contrasted markerless “trace-registration” against fiducial workflows within X-Guide (X-Nav Technologies, Lansdale, PA, USA); and one evaluated an open-sleeve versus closed-sleeve guide design for irrigation optimization. Such variation in planning and registration protocols partly explains the inter-study dispersion observed in the pooled accuracy metrics, but it also enhances the external validity of the synthesis by reflecting the breadth of digital workflows currently available in clinical practice.

Table 2 presents the data extraction table, with each study’s information such as the study design, navigation type, hardware of software used, implant type, number of groups, number of implants, and the three metric data—apical deviation, coronal deviation, and angle deviation.

### 3.3. Risk-of-Bias Findings

The Supplementary Table S2 provides the risk of bias assessment performed for each study, providing a better understanding of the analysis.

Randomized evidence (7 RCTs). Randomisation methods and outcome-data completeness were adequate in every trial. One study achieved an overall “low-risk” judgement, while the remaining six were rated as “some concerns”, chiefly because surgeons could not be blinded and assessor blinding was inconsistently reported.

Non-randomized clinical studies (10 cohorts/case-series). Five investigations were at serious risk of bias, largely owing to residual confounding and outcome assessment by the treating team; the other five were judged as moderate risk. Selection criteria were generally explicit, and follow-up was complete.

In vitro investigations (26 studies). Five studies satisfied all QUIN domains (overall low risk). The other twenty-two were moderate risk, most often because blinding of the examiner measuring implant deviation was absent or unreported. Model standardization, replication, and statistical analysis were usually satisfactory.

**Figure 2:**
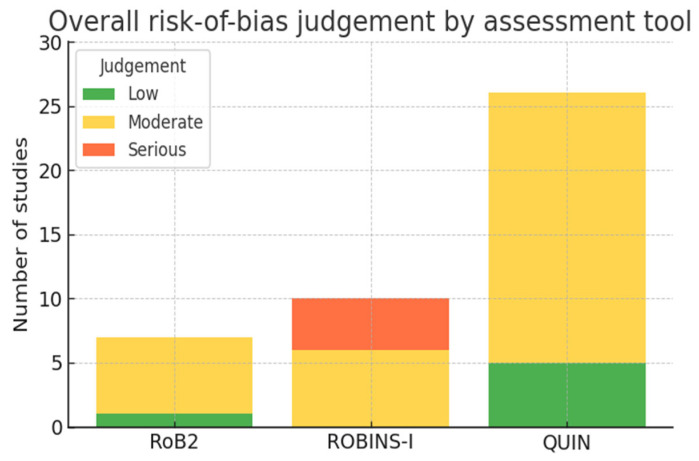
Overall risk-of-bias judgement.

Across all designs, limitations related to blinding and confounding were more common than issues with missing data or selective reporting (see Figure 2 and the Supplementary Table S2).

### 3.4. Surgical Approaches Represented

Robot-guided workflows appeared in 8 studies. Dynamic navigation was the most frequently researched modality (35 papers), predominantly using Navident, Iris-100, or Yizhime platforms. Static stereolithographic guides featured in 24 investigations (proprietary or generic CAD/CAM templates), and FH was retained as a baseline comparator in 21 studies.

### 3.5. Distribution of Implant-Placement Deviations

Implant placement deviation was evaluated comparatively in terms of angular accuracy, apical accuracy, and apical deviation. As the studies are different in their design, the data is organized in grouped charts to illustrate the deviation values from each study spread across different guidance methods. The studies include comparative designs across clinical and in vitro settings. The Data Extraction Table can be analyzed for each study details.

### 3.6. Studies Accuracy

Study-level accuracy across all modalities is presented in the main-text figures. To facilitate detailed inspection without cross-method clutter, we also provide per-modality supplementary plots (robot, dynamic, static, freehand) for angular, apical, and coronal deviations (Supplementary Figures S1–S12), each showing mean ± SD per study arm.

#### 3.6.1. Angular Deviation

- Angle deviation 0–1°

**Figure 3:**
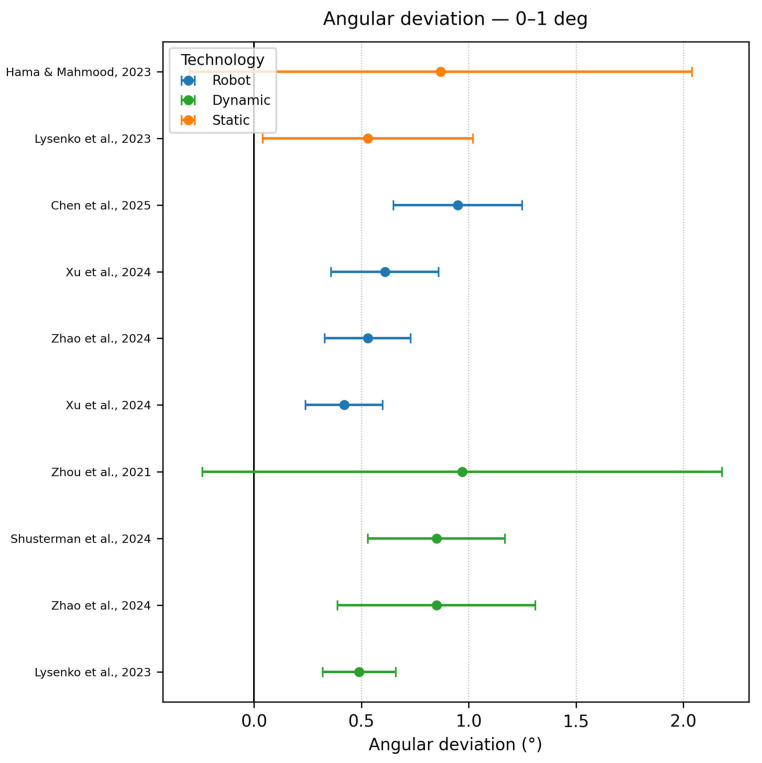
Angle deviation graph in the range of 0–1 degree [3,16,19,31,39,42,50].

Sub-degree control (0–1°, n = 10) is achieved largely by dynamic (four datasets) and robotic workflows (six) [3]. (dynamic AR) reaches 0.70 ± 0.29°. Two static fully guided bench datasets from Lysenko et al., 2023 [31] (0.49 ± 0.17°) and Du et al., 2025 [23] showed the highest point in this range as a robotic study but kept its low deviation at a range of ±0.3. The values regarding this range of precision are represented in Figure 3.

- Angle deviation 1–2°

**Figure 4:**
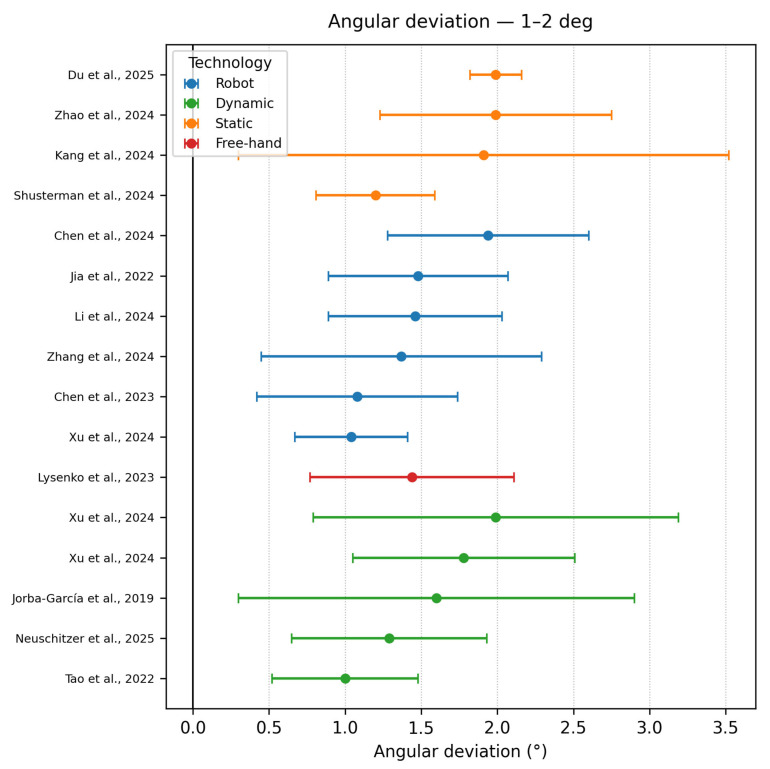
Angle deviation graph—1–2° [3,9,15,16,20,23,31,34,38,39,41,46,48].

Fifteen datasets clustered between 1 and 2°. Again, dynamic navigation dominated, but single values for both static guides (e.g., Zhao et al., 2024 [16] 1.87 ± 0.44°) and freehand (Lysenko et al., 2023 [31], 1.44 ± 0.67°) were present. The dispersion of static data supports the meta-analytic observation by Yu et al., 2023 [51] that angular advantages of dynamic over static guidance average ≈0.8° yet overlap in individual trials. Robotic approaches pose a good dispersion of the accuracy, as shown in Figure 4.

- Angle deviation 2–4°

**Figure 5:**
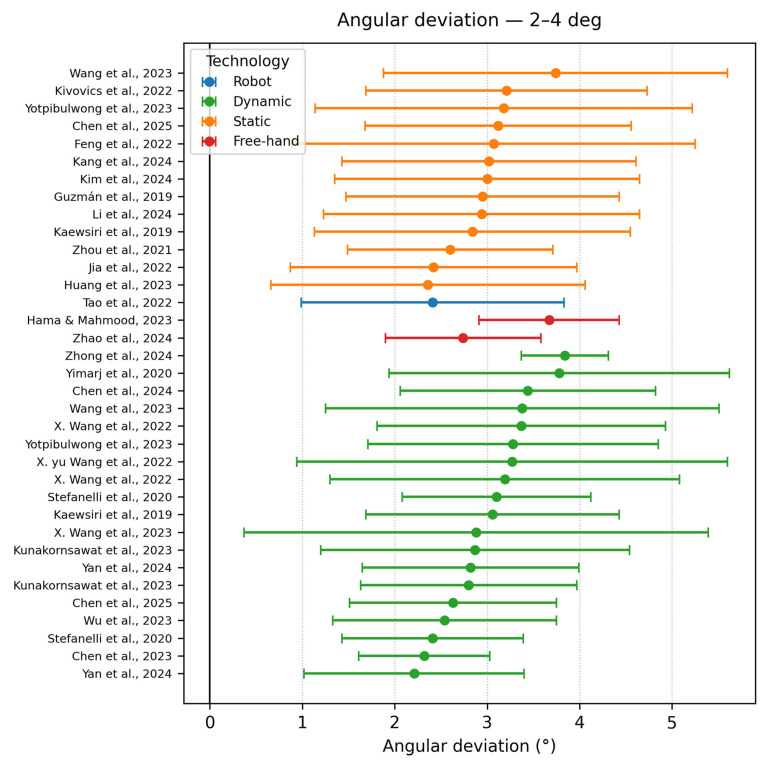
Angle deviation graph—2–4° [9,12,15,19,20,22,23,25,26,27,32,34,35,36,37,38,40,42,43,45,46,47,49].

Twenty-three studies occupied this mid-zone, largely static and dynamic workflows, with only one robotic outlier Tao et al., 2022 [9]. Static guides demonstrated a widespread (2.4–3.9°), reflecting sensitivity to sleeve length and offset reported by Kivovics et al., 2022 [32] and Huang et al., 2023 [43]. Dynamic navigation studies with inexperienced operators (e.g., Kunakornsawat et al., 2023 [26]) also fell into this category, reinforcing that real-time tracking cannot fully compensate for a learning curve. The values are represented in Figure 5.

- Angle deviation ≥ 4°

**Figure 6:**
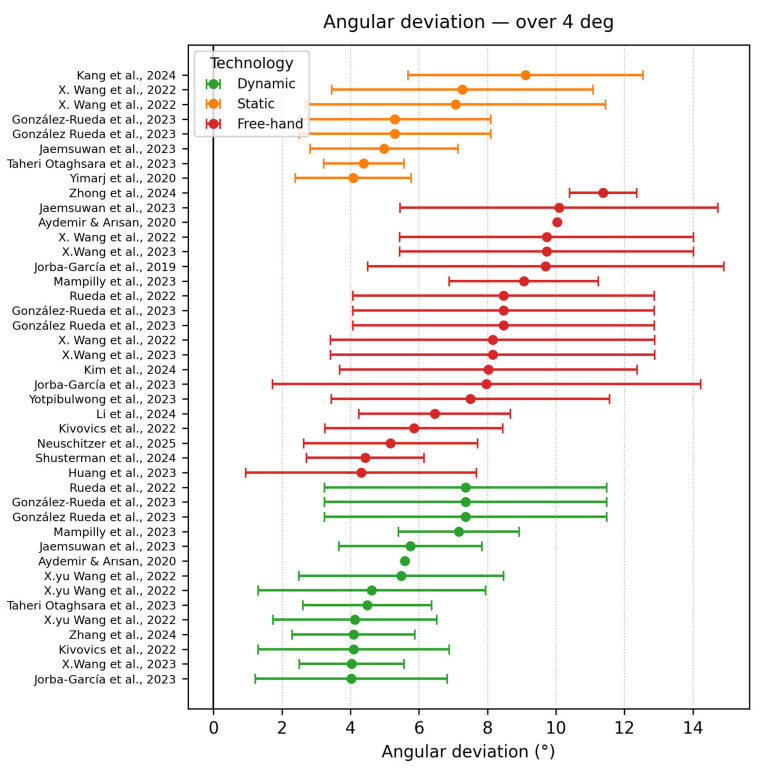
Angle deviation graph ≥ 4° [10,11,15,17,18,20,25,27,29,30,32,33,36,37,39,40,41,43,44,46,48].

Data shown in Figure 6 set exceeded the clinically acceptable 4° threshold, most of them being freehand techniques. Rueda et al., 2022 [44] showed the highest values regarding dynamic navigation due to the novice profile of the surgeon, with experience only in freehand techniques and due to the use of zygomatic implants, five times the length of conventional implants. No robotic study crossed 4°, confirming their capacity to restrain large angular deviations.

#### 3.6.2. Apical Deviation

- Apical accuracy 0–1 mm

**Figure 7:**
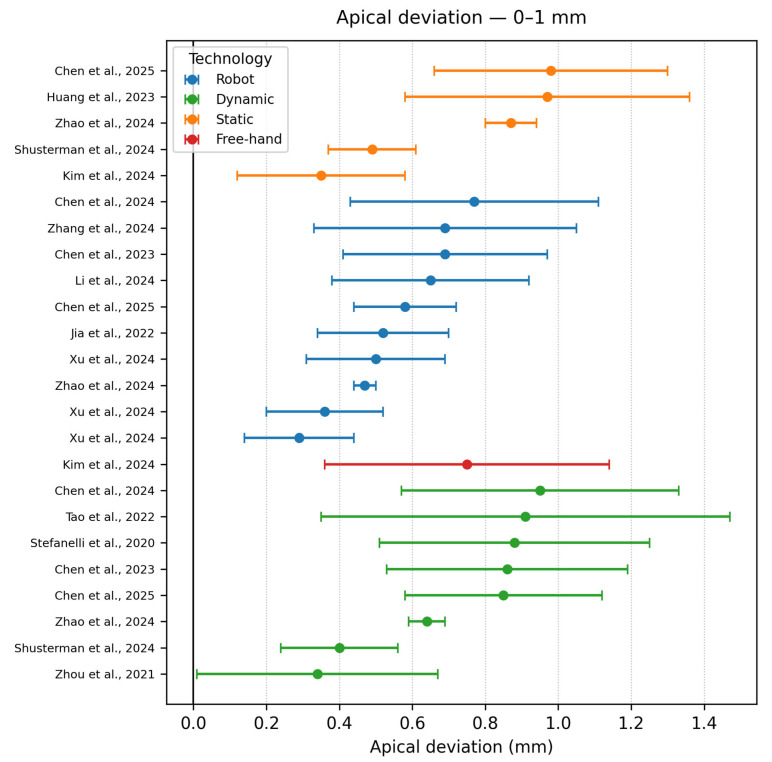
Apical deviation graph—<1 mm [9,10,11,12,15,16,19,20,22,23,26,27,28,32,33,34,35,36,37,38,39,40,41,42,43,45,46,48].

In Figure 7 Eighteen studies, split evenly between dynamic and robotic workflows, achieved < 1 mm apex error. Kim et al., 2024 [37] stands alone as the only static guide study, using a hand-piece-mounted zirconia sleeve, that can lock the drill path to its final diameter, yielding the best results for the static approach, showing that properly designed mechanical constraints can rival with technologically advanced guidance systems. Zhao et al., 2024 [16] shows three studies, robotic (0.47 ± 0.03), dynamic navigation (0.64 ± 0.05) and static navigation (0.87 ± 0.07) on the same polyurethane models, confirming that on-point planning and good standardization can decrease the variability in outcomes. Zhou et al., 2021 [19] reports impressive results in the dynamic approach (0.34 ± 0.33), where postgraduate residents worked with few navigation cases. The data illustrates that true apical precision depends on a solid registration, whether achieved by a passive sleeve, haptic arms, or an optical tracker, while, as shown in the previous papers, experience is associated with greater variability.

- Apical accuracy (1–2 mm)

**Figure 8:**
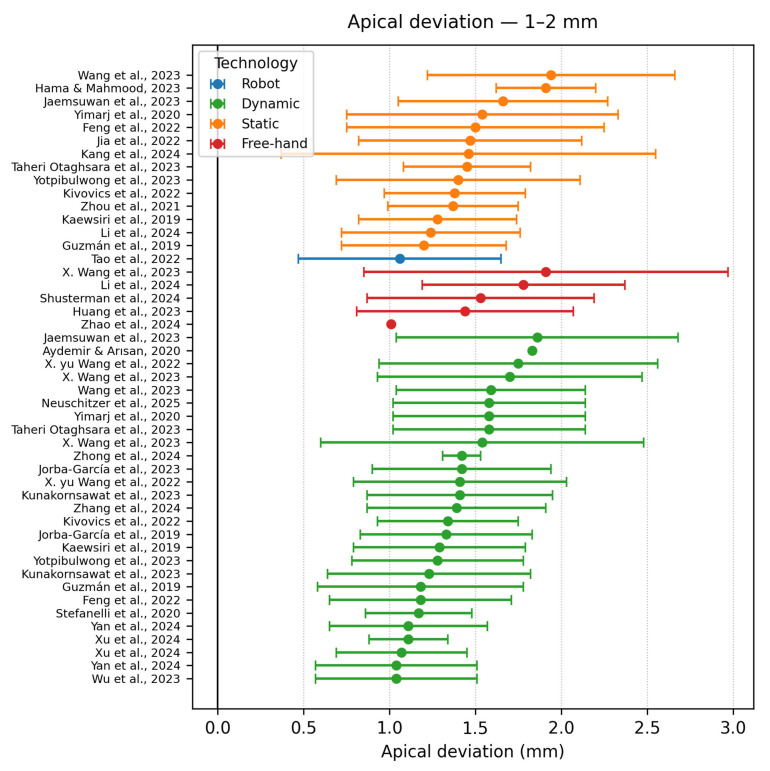
Apical deviation graph—1–2 mm [9,10,12,15,16,19,20,22,25,26,27,28,30,32,33,36,38,39,40,41,42,43,45,46,47,48,49].

Thirty-six datasets lay between 1 and 2 mm (Figure 8). This is the zone where static and dynamic distributions overlap, confirming meta-analytic equivalence for linear metrics. Tao et al., 2022 [9] with a deviation of 1.06 ± 0.59 are the only remaining robotic systems remaining in this range. Stefanelli et al., 2020 [45] reported that dynamic navigation manages to get close to the lower limit of 1 mm, with 3–4 tooth tracing yielding 1.17 ± 0.31 mm and 5–6 tooth tracing 0.88 ± 0.37 mm (pooled mean ~1.01 mm), showing that optical tracking can still provide good outcomes if registration is carefully performed on multiple landmarks. By contrast, Li et al., 2024 [15] static-guide protocol produced a slightly higher global apical deviation of 1.24 ± 0.52 mm, strongly influenced by the fabrication tolerances of the surgical guides. The distribution of freehand techniques is already scarce in this graph, suggesting a majority in the next graph.

- Apical deviation ≥ 2 mm

**Figure 9:**
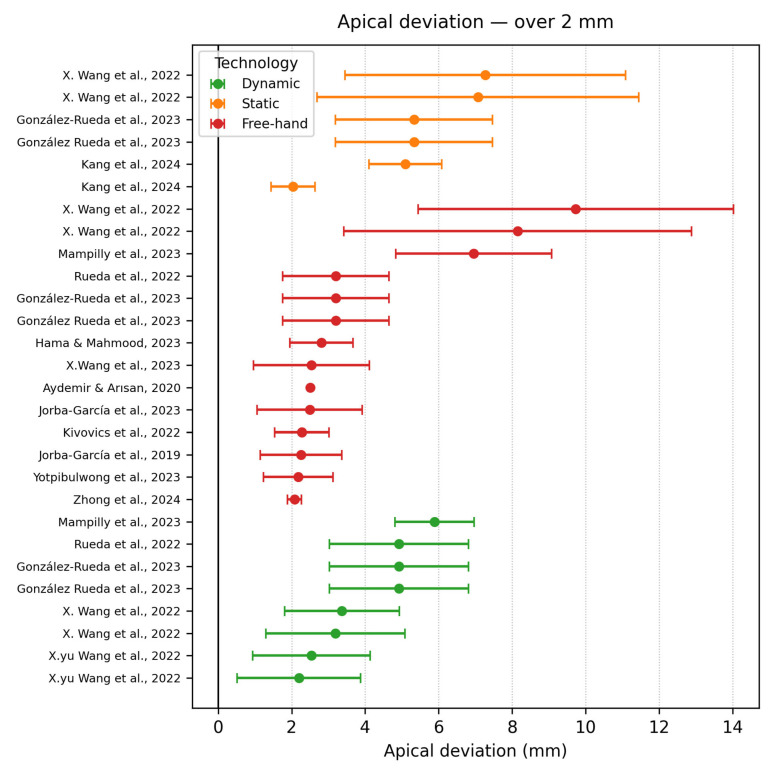
Apical deviation graph—≥2 mm [18,24,25,27,30,32,33,36,42,44,46,48].

Twenty-two datasets exceeded an apical error of 2 mm; their distribution is mainly of static navigation systems and freehand techniques, although a small number of dynamic navigation systems still appear in this range. X. Wang et al., 2022 [29] stands out with two extreme outliers in the same study (shown in Figure 9). The static approach showed that there is a big fluctuation when considering the experience of the surgeon; the experienced group had better results, but with a higher variability, whereas the novice showed a higher mean with a lower deviation (7.27 ± 3.82 for experienced and 7.07 ± 4.38 for novice). Both results arose in dense mandible models. Mampilly et al., 2023 [18] was at the back of the list as well, measuring a mean apical deviation in regard to the dynamic navigation system of 5.89 ± 1.08 mm versus 6.95 ± 2.12 mm for freehand placement. The study highlights that dynamic tracking can be constrained by line-of-sight interruptions and patient movement, that determines the multi-millimetre apex shifts when registration is improper.

#### 3.6.3. Coronal (Platform) Deviation

- Coronal deviation < 1 mm

**Figure 10:**
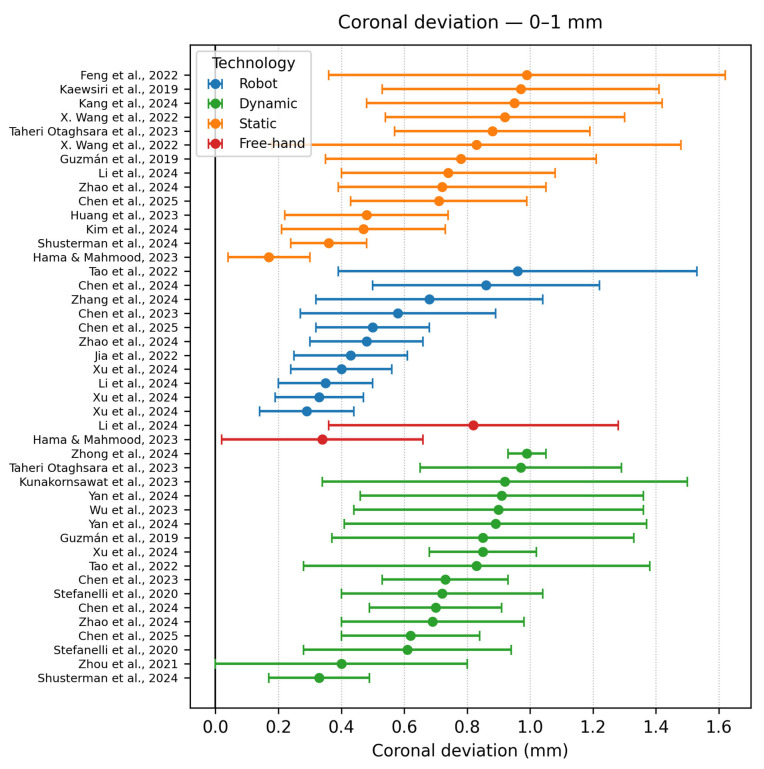
Coronal deviation graph—<1 mm [10,12,15,16,19,20,23,26,29,30,34,35,37,39,42,43,45,46,47,49].

Thirty datasets were <1 mm (Figure 10). Dynamic navigation contributed the largest share, but static guides with rigid metal sleeves [29] were equivalent. Optical tracking is capable of matching robotic precision in straightforward sites, as illustrated by Xu et al., 2024 (in a dynamic clinical cohort, 28 implants planned with Navident and mixed-reality trace registration, showed 0.69 ± 0.25 mm apical deviation [3]) and by the trial of Baoxin [9]. Freehand was rare in this segment; the freehand point (Hama & Mahmood, 2023 [42]) corresponded to a veteran operator in a low-visibility mandible but used extensive surgical stents for alignment.

- Coronal deviation 1–2 mm

**Figure 11:**
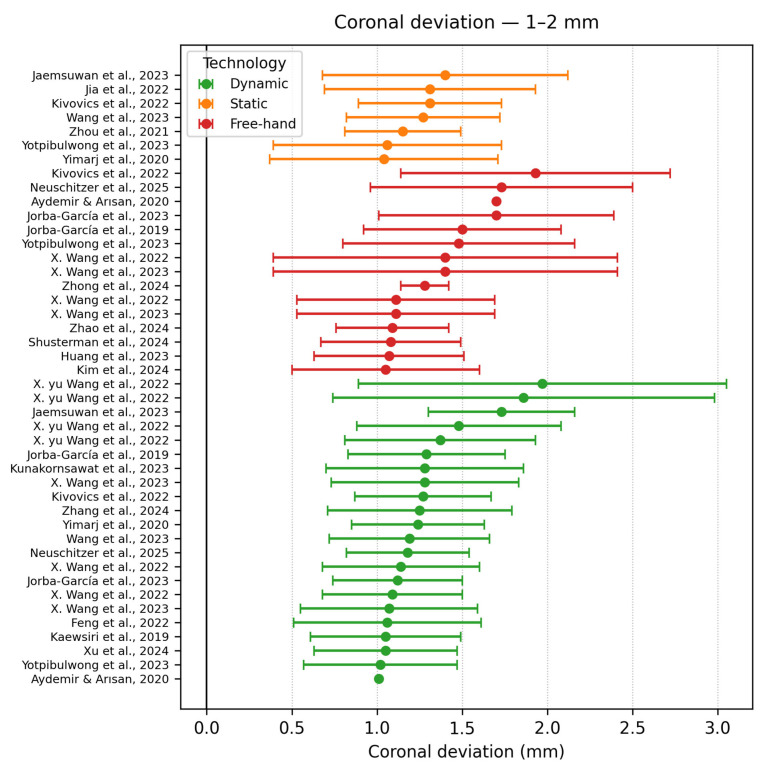
Coronal deviation graph—1–2 mm [9,11,16,22,25,26,27,28,29,30,32,33,36,37,39,40,41,43,48].

In Figure 11, Twenty-seven datasets clustered here, predominantly static and dynamic. Yimarj et al., 2020 [40] (static guide, 1.04 mm but with an SD ± 0.67 mm) reported high variability in their accuracy outcomes, due to the duality of the study. Senior staff placed half of the implants with a deviation around 0.8 mm, whereas residents’ deviations reached up to 1.6 mm. Wang et al., 2022 [29] compared active versus passive infrared navigation systems across a high range of mandible models (64, 704 implants). The learning curve had a profound effect on the outcomes of the study, with the first 12 cases contributing the largest individual errors (>3 mm), widening the SD to ±1.12 mm. The novice-versus-experienced paper by Jorba-Garcia et al.,2019 [48] reinforces that clinically deployed dynamic or static guides typically cluster in 1–1.3 mm range. There is an absence of any robotic systems already in this margin, all of them remaining below the 1 mm threshold (excepting the extent of their SD).

- Coronal deviation ≥ 2 mm

**Figure 12:**
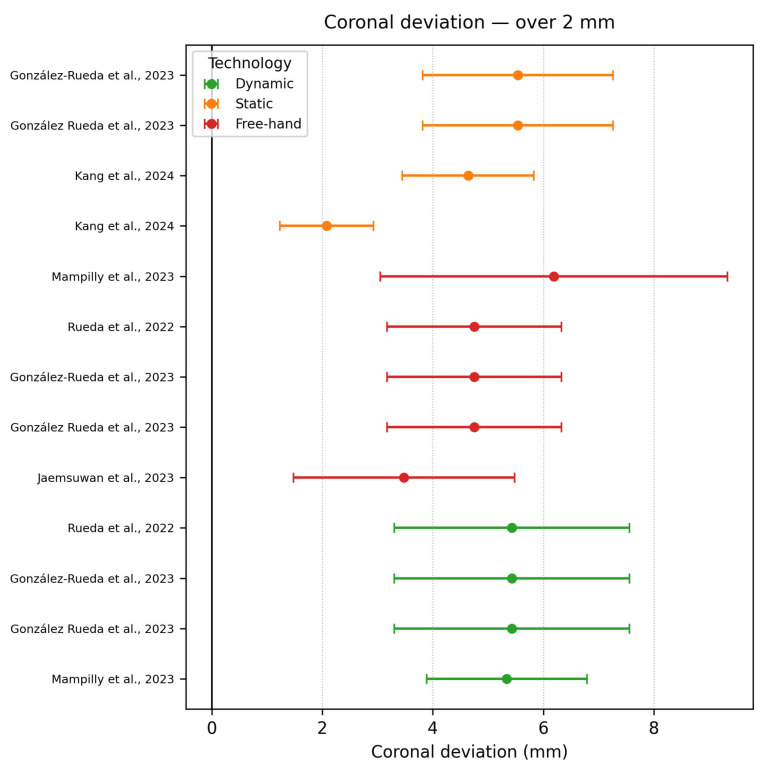
Coronal deviation graph—≥2 mm [17,18,24,28,44,46].

Ten studies consisting of all, but robotic systems exceeded 2 mm. Mampilly et al., 2023 [18] asked five experienced and five novice surgeons to place 4.2 × 10 mm fixtures in three maxillary sites with and without Navident, averaging 5.34 ± 1.45 mm for the navigated arm and 6.19 ± 3.14 mm freehand. Authors tracing this outcome were strongly influenced by the steep learning curve in the successive three-day sessions, where they were still acclimatizing to the hand-eye-screen coordination and the registration error of ≈0.7 mm of the navigation system. González Rueda et al., 2023 [24] reported that high coronal-entry error was determined by the posterior access and the limited visual control and drill chatter in the dense bone, this can be analysed in Figure 12.

## 4. Discussion

### 4.1. Main Findings

Across 43 comparative investigations (2019–2025), every computer-assisted workflow outperformed freehand placement in three-dimensional accuracy. Dynamic systems repeatedly achieved smaller angular deviation than static guides (Kaewsiri et al., 2019 [12]; Taheri Otaghsara et al., 2023 [10]; Yimarj et al., 2020 [40]; Yotpibulwong et al., 2023 [36]), while fully guided static templates approached similar linear precision when rigid metal sleeves or optimized offsets were used (Li et al., 2024 [15]; Kim et al., 2024 [37]). Robotic platforms such as Remebot or task-autonomous cobots yielded sub-millimetre and sub-degree control in laboratory settings (Du et al., 2025 [23]; Xu et al., 2024 [3]), confirming the quantitative hierarchy previously reported by Khaohoen et al., 2024 [1].

### 4.2. Relation to Previous Work

Early attempts to standardize implant positioning relied on two-dimensional intra-oral radiographs and geometric back-projection. Cosola et al., 2021 [52] showed that simple radiographic calibration markers can reconstruct three-dimensional implant position with reasonable precision, highlighting that digital navigation builds on longstanding principles rather than replacing them. Our review builds on these foundational studies by harmonizing accuracy metrics across modalities and integrating newer robotic and zygomatic data under a consistent analytic framework. The accuracy hierarchy observed here mirrors that of Du et al., 2025 [23] and Khan et al., 2024 [5], but contrasts partially with Khaohoen et al., 2024 [1], who reported no significant difference between dynamic and static guidance in clinical trials. This discrepancy likely reflects the inclusion of zygomatic and hybrid workflows in our dataset. Moreover, unlike prior meta-analyses, we did not identify any cadaveric comparative evidence and AI-based systems, exposing a translational gap between laboratory validation and clinical reality

### 4.3. Static vs. Dynamic Navigation

The equivalence of static and dynamic linear metrics seen here mirrors earlier findings (Yu et al., 2023 [51]; Khan et al., 2024 [5]). Differences emerge mainly in angular deviation, where continuous visual feedback enables intra-operative trajectory correction. Studies using Navident and Iris-100 consistently showed a <2° mean error (Aydemir et al., 2020 [33]; Zhou et al., 2021 [19]; Kunakornsawat et al., 2023 [26]), whereas pilot-only static guides drifted toward 3–4° (Kivovics et al., 2022 [32]; Huang et al., 2023 [43]). Hybrid approaches combining static sleeves with real-time feedback improved reproducibility in zygomatic and full-arch cases (Du et al., 2025 [23]; González-Rueda et al., 2023 [17]). These patterns indicate that angular control, more than linear trueness, differentiates dynamic systems.

### 4.4. Dynamic vs. Robotic Guidance

Robotic systems demonstrated narrower deviation ranges (<1 mm, <2°) than manual dynamic navigation under identical conditions (Tao et al., 2022 [9]; Xu et al., 2024 [3]; Zhao et al., 2024 [16]). Chen et al., 2023 [34] confirmed consistent precision in immediate and full-arch placements, while Li et al., 2024 [15] and Zhang et al., 2024 [14] showed reproducibility in anterior and posterior regions. Nevertheless, most robotic data remain in vitro or single-centre clinical, limiting external validity. Current evidence indicates that mechanical constraint and calibration accuracy, rather than autonomous control, account for the observed precision of current robotic workflows.

### 4.5. Strengths and Limitations of the Evidence

Only six of forty-three studies were at low risk of bias (one RCT and five in vitro experiments). Out of the seven randomized clinical trials, six had “some concerns” and only one was “low risk”, mainly due to unavoidable surgeon un-blinding and inconsistent assessor blinding. Among the fourteen non-randomized clinical cohorts/case-series, nine were at serious risk; mainly because accuracy was measured by the treating team or derived retrospectively, while the remaining five were of moderate risk. Of the twenty-six in vitro studies, twenty-two carried a moderate rating (absent or unreported examiner blinding), and five fulfilled all QUIN criteria. These issues may inflate reported advantages and suggest that real-world accuracy gains could be smaller than pooled medians indicate.

Additional heterogeneity arises from varied measurement protocols (single 3-D apex-to-plan distance vs. separate linear and angular metrics) and diverse registration or drill-sequence procedures, particularly within static and dynamic systems. Operator experience was poorly documented; only a handful of studies explored learning-curve effects.

### 4.6. Technology-Specific Considerations

Static templates (s-CAIS). Accuracy depends on guide support (tooth/mucosa/bone-borne), sleeve–drill tolerances, offsets, and multi-step drill stacks. Template stability and irrigation pathways limit performance in posterior or limited-access sites and may influence thermal control.

Dynamic navigation (d-CAIS). Key drivers are registration (fiducials vs. surface matching), line-of-sight for optical tracking, tool calibration (bur swaps, length compensation), and latency. Continuous visual feedback likely underpins the angular advantage observed in several datasets, balanced against cognitive load (screen–field switching) and dependence on tracking quality.

Robot-assisted systems (r-CAIS). Tight clusters likely reflect mechanical constraint (haptic/kinematic limits) and consistent trajectory enforcement. Performance is sensitive to registration accuracy, arm calibration/compensation, stiffness/backlash, and autonomy level (shared control vs. autonomous). In our dataset, clinical r-CAIS evidence draws from relatively few platforms, which constrains causal inference despite encouraging precision.

Accuracy varied with registration and anatomical site. Trace-registration and markerless optical workflows reduced error relative to fiducial approaches (Wu et al., 2023 [20]; Xu et al., 2024 [3]). Posterior maxillae and zygomatic trajectories generated larger angular spread due to restricted access and dense bone (González-Rueda et al.,2023 [24]; Rueda et al., 2022 [44]). Static templates using mucosa-borne supports showed greater variability than tooth- or bone-borne designs (Yimarj et al., 2020 [40]; Lysenko et al., 2023 [31]). Such variability underscores the need for standardized reporting of registration accuracy, sleeve offset, and support type in future studies.

### 4.7. Zygomatic Implants

Zygomatic placement presents unique challenges (long drill path, angulation, and proximity to vital structures), which can magnify systematic and random errors inherent to each guidance modality. Contemporary clinical series using combined static dynamic workflows (Du et al., 2025 [23]) report angular deviations ~2° and coronal global deviations ~1.1–1.3 mm, with apical medians ~1.7 mm, suggesting that hybrid guidance can stabilize coronal control while maintaining acceptable apical accuracy. Within zygomatic cohorts, we observed no clear side-to-side differences, and accuracy appeared comparable across typical implant lengths, although confidence intervals were wide. These findings align with broader trends that dynamic guidance improves 3D control yet remains sensitive to registration fidelity and surgeon experience; combining static sleeves [17] (for initial positioning) with dynamic feedback appears to mitigate drift over long trajectories.

### 4.8. Learning Curve and Training

Wang et al., 2022 [27] conducted an in vitro study with 704 implants using active vs. passive infrared dynamic navigation (Yizhimei) and found that the learning curve approached plateau after approximately 12 cases and converged by 27 placements. Active IR systems reached stable accuracy faster than passive IR systems, with consistently smaller angular and coronal deviations.

Wang et al., 2023 [25] compared freehand, static-guided, and dynamic navigation among novice and experienced surgeons and showed that dynamic navigation significantly reduced angular error compared with other techniques. The accuracy difference between novice and expert users disappeared under dynamic navigation, while procedure time remained longer than freehand but was not affected by experience. Some papers also showed that students using dynamic navigation achieved lower angular deviation in comparison with other approaches, such as freehand (4.9 deg vs. 10.1 deg) and were uniformly satisfied, but the procedure time doubled in initial trials [30]. The influence of gender or gaming familiarity was non-existent based on the finding of Yan et al.,2024 [47].

Kunakornsawat et al., 2023 [26] performed an exploratory randomized trial on novice surgeons using dynamic navigation. The study found that distributed training over several days resulted in faster operative performance and slightly improved coronal accuracy in later sessions compared with same day massed training. Both groups achieved acceptable accuracy levels after the third session.

Education-focused and translational studies indicate rapid proficiency gains with dynamic systems after short, distributed training; template workflows hinge on accurate lab steps and fit checks; robotic systems add setup/calibration time and team coordination. Reporting operator credentials, prior case numbers, and training dose is essential to interpret performance and to generalize across centres.

### 4.9. Clinical Implications

For anatomically constrained or highly aesthetic sites, dynamic navigation already offers a pragmatic accuracy improvement over freehand surgery, especially for inexperienced operators. Static guides remain a cost-effective option for fully edentulous arches or when optical tracking is unavailable. Robotic systems appear most precise but demand higher capital investment and workflow changes. Clinicians should weigh these factors against the modest (≈0.5–1 mm) absolute accuracy benefit.

### 4.10. Translational and Evidence Gaps

Although cadaveric reports exist in the broader CAIS literature, none fulfilled inclusion criteria, mainly for lacking comparative design or full quantitative deviation data, leaving an unvalidated bridge between in vitro precision and realistic anatomy. At the same time, none of the included studies incorporated genuine AI or machine-learning algorithms; all relied on deterministic optical tracking or haptic constraint (Chen et al., 2023 [34]; Zhang et al., 2024 [14]). This confirms that current “robotic intelligence” remains mechanical rather than adaptive. Bridging these gaps requires standardized cadaveric validation and exploration of AI-assisted registration or trajectory optimization.

### 4.11. Future Directions

While the review provides a comprehensive synthesis of the current literature on static, dynamic, and robot-assisted implant systems, several limitations must be acknowledged. A large proportion of the papers included were assessed primarily in vitro or in controlled experimental models, which may not fully replicate clinical variability (soft tissues, patient movement).

Additionally, although all studies reported accuracy outcomes, differences in how these were defined (e.g., 3D apex to plan distance versus separate linear and angular values) may reduce comparability across datasets. The variability in registration protocols, template designs, and drill sequence control especially in static and dynamic systems, although providing high diversity and a better overview of the field, introduces methodological heterogeneity. Future research should prioritize controlled cadaveric trials, standardized 3D deviation metrics, and exploration of AI-assisted registration or adaptive control algorithms.

## 5. Conclusions

This systematic review provides an updated and methodologically harmonized overview of the accuracy of computer-assisted implant surgery (CAIS), integrating evidence from robotic, dynamic, and static systems published between 2019 and 2025. All guided modalities demonstrated superior positional precision compared with freehand placement, confirming the cumulative benefit of computerization in implant surgery. Dynamic and static systems achieved comparable linear accuracy, with dynamic navigation showing a consistent angular advantage. Robotic platforms achieved the tightest accuracy clusters, although clinical data remain limited.

The distinctive contribution of this review lies in its unified comparison across modalities using standardized accuracy definitions, inclusion of hybrid and zygomatic workflows, and synthesis of learning-curve data; dimensions not previously consolidated in a single analysis.

Nowadays static guides and dynamic navigation techniques have reached clinical maturity, whereas robotic systems are still in pioneering age, their continuing development aiming to reduce operator-dependent variability and enhance surgical reproducibility. The clinical evidence base for robotic systems remains relatively limited, with most reported datasets being derived from in vitro or non-randomized studies. This illustrates the need for more high-quality clinical trials to fully assess the impact of robotic assistance in everyday dental surgery practice. Dynamic navigation and robotic systems provide the highest placement accuracy, followed by static guides and freehand drilling. Clinicians should weigh the modest accuracy benefit against equipment cost and training needs, while future well-designed trials are required to confirm these findings.

Clinically, CAIS technologies should be regarded as complementary rather than competitive tools; static guides provide predictable outcomes for routine cases, dynamic systems enable intraoperative adaptability, and robotic assistance offers the potential for maximal reproducibility once validated in larger clinical cohorts.

Future research should prioritize high-quality comparative trials on cadaveric or clinical models, standardized 3D deviation metrics, and exploration of adaptive AI-assisted registration and control. These directions will close the translational gap between laboratory precision and consistent real-world outcomes.

## Figures and Tables

**Table 1:** Search strategy for each database.

PUBMED
((“3D navigation” OR “computer-assisted surgery” OR “robotic-assisted surgery”) AND (“oral surgery” OR “dental surgery” OR “dental implant” OR “implantology” OR “implant”) AND (“static” OR “dynamic” OR “robot assisted” OR “freehand” OR “navigation”) AND (“accuracy” OR “deviation” OR “precision” OR “error”))
Web of Science
TS = ((“3D navigation” OR “computer-assisted surgery” OR “robotic-assisted surgery”) AND (“oral surgery” OR “dental surgery” OR “dental implant” OR “implantology”) AND (“static” OR “dynamic” OR “robot assisted” OR “freehand” OR “navigation”) AND (“accuracy” OR “deviation” OR “precision” OR “error”))
Scopus
TITLE-ABS-KEY “dental implant” AND (“dynamic navigation” OR “static guide” OR “robotic surgery” OR “computer-assisted surgery”) AND (accuracy OR deviation OR precision OR error)) AND SUJAREA (dent) AND PUBYEAR 2019 AND PUBYEAR < 2026

**Table 2:** Data extraction table.

Authors	Study Design	Navigation Type	Software/ Hardware	Implant Type	Group No.	Sample	Apical Deviation	Coronal Deviation	Angle Deviation
Tao et al. [9]	In vitro study	DCAIS RCAIS	BrainLAB CMF v3.0.6 (Brainlab AG, Munich, Germany)	Conv	2	480	RS −1.06 ± 0.59	RS −0.96 ± 0.57	RS −2.41 ± 1.42
							DCAIS −0.91 ± 0.56	DCAIS −0.83 ± 0.55	DCAIS −1 ± 0.48
Pomares Puig et al. [21]	Proof of concept	DCAIS SCAIS Combined	X-Guide	Conv	2	48	Hybrid: 1.42 ± 0.19	Hybrid: 1.25 ± 0.55	Hybrid: 3.74 ± 0.58
Wang et al. [22]	In vitro study	SCAIS DCAIS	Iris-100 (Lizhi YBK Inc., Shanghai, China)	Conv	2	75	DCAIS −1.59 ± 0.55	DCAIS −1.19 ± 0.47	DCAIS −3.38 ± 2.13°
							SCAIS −1.94 ± 0.72	SCAIS −1.27 ± 0.45	SCAIS −3.74 ± 1.86°
Jorba Garcia et al. [11]	Clinical trial	DCAIS Freehand	NaviDent v2.0	Conv	2	22	DCAIS −1.42 ± 0.52	DCAIS −1.12 ± 0.38	DCAIS −4.02 ± 2.80°
							FH −2.49 ± 1.43	FH −1.70 ± 0.69	FH −7.97 ± 6.25°
Zhao et al. [16]	In vitro study	RCAIS, SCAIS DCAIS FH	RCAIS −Remebot (Beijing Bihui Weikang Technology Co., Ltd., Beijing, China) CoDiagnostiX)	Conv	4	120	FH −1.01 ± 0.14	FH −1.09 ± 0.33	FH −2.74 ± 0.84°
							SCAIS −0.87 ± 0.07	SCAIS −0.72 ± 0.33	SCAIS −1.99 ± 0.76°
							DCAIS −0.64 ± 0.05	DCAIS −0.69 ± 0.29	DCAIS −0.85± 0.46
							RCAIS −0.47 ± 0.03	RCAIS −0.48 ± 0.18	RCAIS −0.53 ±0.20°
Du et al. [23]	In vitro study	Hybrid (s-CAIS + d-CAIS)	Yizhimei (Digital-Care Medical Technology Co., Ltd., Suzhou, China)	Zygo	2	22	Hybrid: 1.67 mm	Hybrid: 1.13 ± 0.09	Hybrid: 1.99 ± 0.17
Taheri Otaghsara et al. [10]	In vitro study	SCAIS DCAIS	DENACAM (Mininavident AG, Liestal, Switzerland) coDiagnostiX v10.4.2	Conv	2	40	SCAIS −1.45 ± 0.37	SCAIS −0.88 ± 0.31	SCAIS −4.39 ± 1.17°
							DCAIS −1.58 ± 0.56	DCAIS −0.97 ± 0.32	DCAIS −4.49 ± 1.88°
González Rueda et al. [24]	Clinical trial	SCAIS DCAIS FREEHAND	NaviDent	Zygo	3	60	SCAIS −5.33 ± 2.14	SCAIS −5.54 ± 1.72	SCAIS −5.30 ± 2.80
							DCAIS −4.92 ± 1.89	DCAIS −5.43 ± 2.13	DCAIS −7.36 ± 4.12
							FH −3.20 ± 1.45	FH −4.75 ± 1.58	FH −8.47 ± 4.40
Xu et al. [3]	In vitro study	RCAIS −ARG, PRG, SRG DCAIS −ADG, PDG	DCarer (Dcarer Medical Technology Co., Ltd., Suzhou, China) Active Implant Robot (Yekebot Technology Co, Ltd., Beijing, China) Polaris (NDI Inc., Waterloo, ON, Canada)	Conv	2	216	DCAIS −1.11 ± 0.23 DCAIS −1.07 ± 0.38	DCAIS −0.85 ± 0.17 DCAIS −1.05 ± 0.42	DCAIS −1.78 ± 0.73 DCAIS −1.99 ± 1.20
							RCAIS −0.29 ± 0.15 RCAIS −0.50 ± 0.19 RCAIS −0.36 ± 0.16	RCAIS −0.29 ± 0.15 RCAIS −0.40 ± 0.16 RCAIS −0.33 ± 0.14	RCAIS −0.61 ± 0.25 RCAIS −1.04 ± 0.37 RCAIS −0.42 ± 0.18
X.Wang et al. [25]	Comparison study	FREEHAND DCAIS	NaviDent	Conv	2	72	FH −1.91 ± 1.06 FH −2.54 ± 1.58	FH −1.11 ± 0.58 FH −1.40 ± 1.01	FH −9.73 ± 4.29 FH −8.15 ± 4.73
							DCAIS −1.70 ± 0.77 DCAIS −1.54 ± 0.94	DCAIS −1.28 ± 0.55 DCAIS −1.07 ± 0.52	DCAIS −4.03 ± 1.53 DCAIS −2.88 ± 2.51
Kunakornsawat et al. [26]	Clinical trial	DCAIS	Iris-100 (EPED Inc., Taipei, Taiwan)	Conv	2	144	DG −1.23 ± 0.59	DG −0.92 ± 0.58	DG −2.80 ± 1.17
							MG −1.41 ± 0.54	MG −1.28 ± 0.58	MG −2.87 ± 1.67
X.yu Wang et al. [27]	In vitro study	DCAIS—Active and Passive M-PBR F-PBR	Yizhime (Digital-Care Medical Technology Co., Ltd., Suzhou, China) Iris ((EPED Inc., Taipei, Taiwan))	Conv	4	704	Active −1.75 ± 0.81 Passive −2.20 ± 1.68	Active −1.48 ± 0.60 Passive −1.86 ± 1.12	Active −4.13 ± 2.39 Passive −4.62 ± 3.32
							F-PBR −2.54 ± 1.60 M-PBR −1.41 ± 0.62	F-PBR −1.97 ± 1.08 M-PBR −1.37 ± 0.56	F-PBR −5.48 ± 2.99 M-PBR −3.27 ± 2.33
Jaemsuwana et al. [28]	Non-randomized prospective study	FREEHAND SCAIS DCAIS	Iris-100 coDiagnostiX v9.7	Conv	3	60	FH −3.60 ± 2.11 SCAIS −1.66 ± 0.61 DCAIS −1.86 ± 0.82	FH −3.48 ± 2.00 SCAIS −1.40 ± 0.72 DCAIS −1.73 ± 0.43	FH −10.09 ± 4.64 SCAIS −4.98 ± 2.16) DCAIS −5.75 ± 2.09
X. Wang et al. [29]	In vitro study	FREEHAND SCAIS DCAIS	X-Guide X-Clip (X-Nav Technologies LLC, Lansdale, PA, USA)	Conv	3	72	FH – EXP −9.73 ± 4.29 NOV −8.15 ± 4.73	FH – EXP −1.11 ± 0.58 NOV −1.40 ± 1.01	FH – EXP −9.73 ± 4.29 NOV −8.15 ± 4.73
							SCAIS – EXP −7.27 ± 3.82 NOV −7.07 ± 4.38	SCAIS – EXP −0.83 ± 0.65 NOV −0.92 ± 0.38	SCAIS – EXP −7.27 ± 3.82 NOV −7.07 ± 4.38
							DCAIS – EXP −3.37 ± 1.56 NOV −3.19 ± 1.89	DCAIS – EXP −1.09 ± 0.41 NOV −1.14 ± 0.46	DCAIS – EXP −3.37 ± 1.56 NOV −3.19 ± 1.89
González-Rueda et al. [17]	In vitro study	SCAIS DCAIS AR SYSTEM FREEHAND	NaviDent	Zygo	4	80	SCAIS −5.33 ± 2.14	SCAIS −5.54 ± 1.72	SCAIS −5.30 ± 2.80°
							DCAIS −4.92 ± 1.89	DCAIS −5.43 ± 2.13	DCAIS −7.36 ± 4.12°
							AR SYSTEM −4.88 ± 1.54	AR SYSTEM −5.64 ± 1.11	AR SYSTEM −9.60 ± 4.25°
							FH −3.20 ± 1.45	FH −4.75 ± 1.58	FH −8.47 ± 4.40°
Zhong et al. [30]	Case study	FREEHAND DCAIS	DHC DI2 (Dcarer Medical Technology Co., Ltd., Suzhou, China)	Conv	2	20	FH −2.08 ± 0.19	FH −1.28 ± 0.14	FH −11.38 ± 0.98°
							DCAIS −1.42 ± 0.11	DCAIS −0.99 ± 0.06	DCAIS −3.84 ± 0.47°
Zhou et al. [19]	In vitro study	SCAIS DCAIS	Yizhimei	Conv	2	80	SCAIS −1.37 ± 0.38	SCAIS −1.15 ± 0.34	SCAIS −2.60 ± 1.11°
							DCAIS −0.34 ± 0.33	DCAIS −0.40 ± 0.41	DCAIS −0.97 ± 1.21°
Wu et al. [20]	Clinical analysis	Manual/ Automatic DCAIS	DHC-DI2 Dcarer	Conv	2	58	Manual:0.99 ± 0.48	Manual:0.91 ± 0.45	Manual:2.82 ± 1.17°
							Auto: 1.11 ± 0.46	Auto: 2.21 ± 1.19°	Auto: 2.21 ± 1.19°
Lysenko et al. [31]	In vitro study	SCAIS DCAIS FREEHAND	OptiTrack 13 Prime Exoplan v3.0 (Exocad GmbH, Darmstadt, Germany)	Conv	3	63	NR	NR	SCAIS: 0.53 ± 0.49°
									DCAIS: 0.49± 0.17°
									FH: ≈ 1.44 ± 0.67°
Kivovics et al. [32]	In vitro study	AR-DCAIS SCAIS FREEHAND	Innooral System (Innoimplant Ltd., Budapest, Hungary) Magic Leap One (Magic Leap Inc., Miami, FL, USA)	Conv	3	48	AR-DCAIS −1.34 ± 0.41	AR-DCAIS −1.27 ± 0.40	AR-DCAIS −4.09 ± 2.79
							SCAIS −1.38 ± 0.41	SCAIS −1.31 ± 0.42	SCAIS −3.21 ± 1.52
							FH −2.28 ± 0.74	FH −1.93 ± 0.79	FH −5.85 ± 2.60
Aydemir & Arısan [33]	Clinical trial	DCAIS FREEHAND	NaviDent v1.3 EvaluNav (ClaroNav Technology Inc. Toronto, ON, Canada)	Conv	2	92	DCAIS −1.83 (±0.12)	DCAIS −1.01 (±0.07)	DCAIS- 5.59 (±0.39)
							FH −2.51 (±0.21)	FH −1.70 (±0.13)	FH −10.04 (±0.83)
Mampilly et al. [18]	In vitro study	DCAIS FREEHAND	NaviDent Evalunav	Conv	10	60	DCAIS −5.89 ± 1.08	DCAIS −5.34 ± 1.45 mm	DCAIS −7.16 ± 1.76
							FH −6.95 ± 2.12	FH −6.19 ± 3.14	FH −9.06 ± 2.18
Chen et al. [13]	In vitro study	RCAIS DCAIS	Remebot Yizhime	Conv	2	80	RCAIS −0.77 ± 0.34	RCAIS −0.86 ± 0.36	RCAIS −1.94 ± 0.66°
							DCAIS −0.95 ± 0.38	DCAIS −0.70 ± 0.21	DCAIS −3.44 ± 1.38°
Chen et al. [34]	In vitro study	RCAIS DCAIS	THETA Robot (Hangzhou Jianjia Robot Co., Ltd., Hangzhou, China) Yizhimei	Conv	2	20	RCAIS: 0.69 ± 0.28	RCAIS −0.58 ± 0.31	RCAIS −1.08 ± 0.66
							DCAIS: 0.86 ± 0.33	DCAIS −0.73 ± 0.20	DCAIS −2.32 ± 0.71
Feng et al. [35]	Prospective study	DCAIS SCAIS	Dcarer	Conv	2	40	SCAIS −1.50 ± 0.75	SCAIS −0.99 ± 0.63	SCAIS −3.07 ± 2.18
							DCAIS −1.18 ± 0.53	DCAIS −1.06 ± 0.55	DCAIS −3.23 ± 1.67
Yotpibulwong et al. [36]	Clinical trial	SCAIS DCAIS FREEHAND	Iris-100 coDiagnostiX v9.7	Conv	4	120	SD −0.75 ± 0.57	SD −0.62 ± 0.50	SD −1.24 ± 1.41
							S −1.40 ± 0.71	S −1.06 ± 0.67	S −3.18 ± 2.04
							D −1.28 ± 0.50	D −1.02 ± 0.45	D −3.28 ± 1.57
							FH −2.18 ± 0.9	FH −1.48 ± 0.68	FH −7.50 ± 4.06
Kim et al. [37]	Clinical study	SCAIS FREEHAND	Simple Guide Device—custom-designed Osstem TS III (Osstem Implant Co., Ltd., Seoul, Republic of Korea)	Conv	4	124	FH: 0.75 ± 0.39	FH: 1.05 ± 0.55	FH: 8.03 ± 4.34
							SCAIS: 0.35 ± 0.23	SCAIS: 0.47 ± 0.26	SCAIS: 3.00 ± 1.65
Zhang et al. [14]	Retrospective study	DCAIS RCAIS	Remebot (Beijing Baihui Weikang Technology Co., Ltd., Beijing, China) Dcarer v1.0	Conv	2	124	DCAIS: 1.39 ± 0.52	DCAIS: 1.25 ± 0.54	DCAIS: 4.09 ± 1.79
							RCAIS: 0.69 ± 0.36	RCAIS: 0.68 ± 0.36	RCAIS: 1.37 ± 0.92
Jia et al. [38]	Retrospective study	RCAIS SCAIS	ADIR (YakeRobot Technology Ltd., Xi’an, China) coDiagnostiX	Conv	2	60	RCAIS −0.52 ± 0.18	RCAIS −0.43 ± 0.18	RCAIS −1.48 ± 0.59
							SCAIS −1.47 ± 0.65	SCAIS −1.31 ± 0.62	SCAIS −2.42 ± 1.55
Li et al. [15]	Retrospective study	FREEHAND SCAIS RCAIS	Remebot	Conv	3	106	FH −1.78 ± 0.59	FH −0.82 ± 0.46	FH −6.46 ± 2.21
							SCAIS −1.24 ± 0.52	SCAIS −0.74 ± 0.34	SCAIS −2.94 ± 1.71
							RCAIS −0.65 ± 0.27	RCAIS −0.35 ± 0.15	RCAIS −1.46 ± 0.57
Shusterman et al. [39]	In vitro study	MR-DCAIS SCAIS FREEHAND	HoloLens 2 (Microsoft Corp., Redmond, WA, USA) ANNA v1.8.5 (MARS Dental AI, Haifa, Israel) M-guide v2.19.0 (M-Soft, MIS Implant Technologies Ltd., HaZafon, Israel)	Conv	3	135	DCAIS −0.40 ± 0.16	DCAIS −0.33 ± 0.16	DCAIS −0.85 ± 0.32
							SCAIS −0.49 ± 0.12	SCAIS −0.36± 0.12	SCAIS −1.20 ± 0.39
							FH −1.53 ± 0.66	FH −1.08 ± 0.41	FH −4.43 ± 1.72°
Yimarj et al. [40]	Clinical trial	SCAIS DCAIS	Iris-100 coDiagnostiX v9.7	Conv	2	60	SCAIS −1.54 ± 0.79	SCAIS −1.04 ± 0.67	SCAIS −4.08 ± 1.69
							DCAIS −1.58 ± 0.56	DCAIS −1.24 ± 0.39	DCAIS −3.78 ± 1.84
Neuschitzer et al. [41]	In vitro study	DCAIS Freehand	NaviDent	Conv	2	18	FH −1.39 ± 0.74	FH −1.73 ± 0.77	FH −5.17 ± 2.54
							DCAIS −1.58 ± 0.56	DCAIS −1.18 ± 0.36	DCAIS −1.29 ± 0.64
Hama & Mahmood [42]	In vitro study	SCAIS FREEHAND	CoDiagnostiX	Conv	2	60	FH: 2.81 ± 0.86	FH: 0.34 ± 0.32	FH: 3.67 ± 0.76
							sCAIS: 1.91 ± 0.29	sCAIS: 0.17 ± 0.13	sCAIS: 0.87 ± 1.17
Huang et al. [43]	Clinical study	SCAIS FREEHAND	Materialise Magics	Conv	2	48	FH −1.44 ± 0.63	FH −1.07 ± 0.44	FH −4.31 ± 3.37
							SCAIS −0.97 ± 0.39	SCAIS −0.48 ± 0.26	SCAIS −2.36 ± 1.70
Rueda et al. [44]	In vitro study	DCAIS FREEHAND	NaviDent EvaluNav	Zygo	2	39	FH −3.20 ± 1.45	FH −4.75 ± 1.58	FH −8.47 ± 4.40
							DCAIS −4.92 ± 1.89	DCAIS −5.43 ± 2.13	DCAIS −7.36 ± 4.12
Stefanelli et al. [45]	Case study	DCAIS	NaviDent EvaluNav	Conv	2	77	Group A −1.17 ± 0.31	Group A −0.72 ± 0.32	Group A −3.10 ± 1.02
							Group B −0.88 ± 0.37	Group B −0.61 ± 0.33	Group B −2.41 ± 0.98
Kang et al. [46]	Retrospective study	SCAIS	3Shape Implant Studio (3Shape A/S, Copenhagen, Denmark)	Conv	3	62	SCAIS −5.09 ± 0.99 SCAIS- 2.04 ± 0.60 SCAIS −1.46 ± 1.09	SCAIS −4.64 ± 1.19 SCAIS −2.08 ± 0.85 SCAIS −0.95 ± 0.47	SCAIS −9.11 ± 3.43 SCAIS −1.91 ± 1.61 SCAIS −3.02 ± 1.59
Yan et al. [47]	In vitro study	DCAIS	Yizhime	Conv	2	208	DCAIS: 1.04 ± 0.47 DCAIS: 1.11 ± 0.46	DCAIS: 0.91 ± 0.45 DCAIS: 0.89 ± 0.48	CAIS: 2.21 ± 1.19 DCAIS: 2.82 ± 1.17
Jorba-Garcia et al. [48]	In vitro study	DCAIS Freehand	NaviDent EvaluNav	Conv	2	36	FH: 2.26 ± 1.11	FH: 1.50 ± 0.58	FH: 9.7 ± 5.2
							DCAIS: 1.33 ± 0.50	DCAIS: 1.29 ± 0.46	DCAIS: 1.6 ± 1.3
Guzman et al. [49]	In vitro study	SCAIS DCAIS	CoDiagnostiX NaviDent	Conv	2	40	SCAIS: 1.20 ± 0.48	SCAIS: 0.78 ± 0.43	SCAIS: 2.95 ± 1.48
							DCAIS: 1.18 ± 0.60	DCAIS: 0.85 ± 0.48	DCAIS: 4.00 ± 1.41
Chen et al. [50]	In vitro study	SCAIS DCAIS RCAIS	coDiagnostiX v10.4.2 Optitrack sa-RASS (Hangzhou Jianjia Medical Technology Co., Ltd., Hangzhou, China) Prototype Robot	Conv	3	90	SCAIS: 0.98 ± 0.32	SCAIS: 0.71 ± 0.28	SCAIS: 3.12 ± 1.44
							DCAIS: 0.85 ± 0.27	DCAIS: 0.62 ± 0.22	DCAIS: 2.63 ± 1.12
							RCAIS: 0.58 ± 0.14	RCAIS: 0.50 ± 0.18	RCAIS: 0.95 ± 0.30
Kaewsiri et al. [12]	Randomized controlled clinical trial	SCAIS DCAIS	CoDiagnostiX v9.7 IRIS-100	Conv	2	48	SCAIS: 1.28 ± 0.46	SCAIS: 0.97 ± 0.44	SCAIS: 2.84 ± 1.71
							DCAIS: 1.29 ± 0.50	DCAIS: 1.05 ± 0.44	DCAIS: 3.06 ± 1.37

## Data Availability

The original contributions presented in this study are included in the article. Further inquiries can be directed to the corresponding authors.

## Supplementary Materials

The following supporting information can be downloaded at: https://www.mdpi.com/article/10.3390/dj13110537/s1, Figure S1: angular_dynamic, Figure S2: angular_dynamic; Figure S3: angular_dynamic, Figure S4: angular_dynamic, Figure S5: angular_dynamic, Figure S6: angular_dynamic, Figure S7: angular_dynamic, Figure S8: angular_dynamic, Figure S9: angular_dynamic, Figure S10: angular_dynamic, Figure S11: angular_dynamic, Figure S12: angular_dynamic. Table S1: PRISMA_2020_Checklist; Table S2: Supplementary Table S2.
