# Supplementary material for: Accuracy of Navigation and Robot-Assisted Systems for Dental Implant Placement: A Systematic Review
Source: Dent J (Basel). 2025 Nov 14;13(11):537. doi: 10.3390/dj13110537 (PMC12651058; doi:10.3390/dj13110537)
Supplement: Supplementary file 1 [file dentistry-13-00537-s001.zip › dentistry-3824564-newsupplementary/Supplementary table S1-PRISMA 2020 Checklist.docx]

| **Section and Topic** | **Item #** | **Checklist item** | **Location where item is reported** |
| --- | --- | --- | --- |
| **TITLE** | | |  |
| Title | 1 | Identify the report as a systematic review. | Title page — identified as a systematic review (‘Systematic Review’ in title). |
| **ABSTRACT** | | |  |
| Abstract | 2 | See the PRISMA 2020 for Abstracts checklist. | Abstract — structured summary. |
| **INTRODUCTION** | | |  |
| Rationale | 3 | Describe the rationale for the review in the context of existing knowledge. | Introduction §1 (Rationale). |
| Objectives | 4 | Provide an explicit statement of the objective(s) or question(s) the review addresses. | Introduction §1 (Aim at end). |
| **METHODS** | | |  |
| Eligibility criteria | 5 | Specify the inclusion and exclusion criteria for the review and how studies were grouped for the syntheses. | §2.3 Eligibility Criteria and §2.8 Synthesis Methods (grouping by CAIS modality: r-CAIS, d-CAIS, s-CAIS, FH) |
| Information sources | 6 | Specify all databases, registers, websites, organisations, reference lists and other sources searched or consulted to identify studies. Specify the date when each source was last searched or consulted. | §2.5 Search Strategy — PubMed/MEDLINE, Scopus, Web of Science (coverage 2019–2025; last search April 2025) |
| Search strategy | 7 | Present the full search strategies for all databases, registers and websites, including any filters and limits used. | §2.5 + **Table 1** (full Boolean syntax for PubMed, WoS, Scopus) |
| Selection process | 8 | Specify the methods used to decide whether a study met the inclusion criteria of the review, including how many reviewers screened each record and each report retrieved, whether they worked independently, and if applicable, details of automation tools used in the process. | Methods §2.6 Study-selection — two reviewers in Rayyan; PRISMA flow. |
| Data collection process | 9 | Specify the methods used to collect data from reports, including how many reviewers collected data from each report, whether they worked independently, any processes for obtaining or confirming data from study investigators, and if applicable, details of automation tools used in the process. | §2.4 — dual independent extraction; discrepancies resolved by discussion; author contact if necessary |
| Data items | 10a | List and define all outcomes for which data were sought. Specify whether all results that were compatible with each outcome domain in each study were sought (e.g. for all measures, time points, analyses), and if not, the methods used to decide which results to collect. | §2.6 Types of Outcome Measures — platform, apex, depth, and angular deviation (in mm and degrees) |
|  | 10b | List and define all other variables for which data were sought (e.g. participant and intervention characteristics, funding sources). Describe any assumptions made about any missing or unclear information. | §2.2 PICOS + §2.4 (data on design, registration, conversions, learning curve, other variables) |
| Study risk of bias assessment | 11 | Specify the methods used to assess risk of bias in the included studies, including details of the tool(s) used, how many reviewers assessed each study and whether they worked independently, and if applicable, details of automation tools used in the process. | §2.7 Risk-of-Bias Assessment — RoB 2, ROBINS-I, and QUIN; two independent reviewers + third adjudicator; Excel-based traffic-light figure (Fig. 2) |
| Effect measures | 12 | Specify for each outcome the effect measure(s) (e.g. risk ratio, mean difference) used in the synthesis or presentation of results. | §2.6 (Mean ± SD; descriptive comparison only, no pooled effect size) |
| Synthesis methods | 13a | Describe the processes used to decide which studies were eligible for each synthesis (e.g. tabulating the study intervention characteristics and comparing against the planned groups for each synthesis (item #5)). | §2.8 Synthesis Methods + §3.2/§3.6 Results (grouped by modality and outcome type) |
|  | 13b | Describe any methods required to prepare the data for presentation or synthesis, such as handling of missing summary statistics, or data conversions. | §2.4 (data conversions: RMS→mean/SD; vector-norm computation) |
|  | 13c | Describe any methods used to tabulate or visually display results of individual studies and syntheses. | §3.2 Table 2; §3.6 Figures 3–12 + Supplementary Figures S1–S12 |
|  | 13d | Describe any methods used to synthesize results and provide a rationale for the choice(s). If meta-analysis was performed, describe the model(s), method(s) to identify the presence and extent of statistical heterogeneity, and software package(s) used. | §2.8 and §3.6 (no meta-analysis; descriptive synthesis due to heterogeneity) |
|  | 13e | Describe any methods used to explore possible causes of heterogeneity among study results (e.g. subgroup analysis, meta-regression). | §3.6 qualitative exploration by modality and operator experience (no subgroup statistics) |
|  | 13f | Describe any sensitivity analyses conducted to assess robustness of the synthesized results. | Not conducted (no meta-analysis or sensitivity tests) |
| Reporting bias assessment | 14 | Describe any methods used to assess risk of bias due to missing results in a synthesis (arising from reporting biases). | §2.7 — addressed within RoB domains; funnel plots not applicable |
| Certainty assessment | 15 | Describe any methods used to assess certainty (or confidence) in the body of evidence for an outcome. | Not conducted (no GRADE assessment) |
| **RESULTS** | | |  |
| Study selection | 16a | Describe the results of the search and selection process, from the number of records identified in the search to the number of studies included in the review, ideally using a flow diagram. | §3.1 Identification and Screening + **Fig. 1** (PRISMA 2020 flow: 843→43) |
|  | 16b | Cite studies that might appear to meet the inclusion criteria, but which were excluded, and explain why they were excluded. | §3.1 Results and Fig. 1 (summarized exclusion reasons); full log available on request |
| Study characteristics | 17 | Cite each included study and present its characteristics. | §3.2 Study Range and Characteristics + **Table 2** (data-extraction table for 43 studies) |
| Risk of bias in studies | 18 | Present assessments of risk of bias for each included study. | §3.3 Risk-of-Bias Findings + **Fig. 2** + Supplementary Table S1 |
| Results of individual studies | 19 | For all outcomes, present, for each study: (a) summary statistics for each group (where appropriate) and (b) an effect estimate and its precision (e.g. confidence/credible interval), ideally using structured tables or plots. | §3.6 Results (Angular, Apical, Coronal Deviation graphs and text) + Figs. 3–12 |
| Results of syntheses | 20a | For each synthesis, briefly summarise the characteristics and risk of bias among contributing studies. | §3.2 and §3.3 (summary by modality and RoB levels) |
|  | 20b | Present results of all statistical syntheses conducted. If meta-analysis was done, present for each the summary estimate and its precision (e.g. confidence/credible interval) and measures of statistical heterogeneity. If comparing groups, describe the direction of the effect. | Not applicable (no meta-analysis performed) |
|  | 20c | Present results of all investigations of possible causes of heterogeneity among study results. | §3.6 (qualitative discussion of dispersion, operator experience, model type) |
|  | 20d | Present results of all sensitivity analyses conducted to assess the robustness of the synthesized results. | Not applicable (no sensitivity analyses). |
| Reporting biases | 21 | Present assessments of risk of bias due to missing results (arising from reporting biases) for each synthesis assessed. | Not applicable (no quantitative synthesis to assess small-study effects). |
| Certainty of evidence | 22 | Present assessments of certainty (or confidence) in the body of evidence for each outcome assessed. | Not conducted (no GRADE / certainty). |
| **DISCUSSION** | | |  |
| Discussion | 23a | Provide a general interpretation of the results in the context of other evidence. | Discussion §4.1–4.2 (interpretation vs prior evidence). |
|  | 23b | Discuss any limitations of the evidence included in the review. | §4.5 Strengths and Limitations of the Evidence |
|  | 23c | Discuss any limitations of the review processes used. | §4.5 (last paragraph — limitations due to unblinded assessors and heterogeneous protocols |
|  | 23d | Discuss implications of the results for practice, policy, and future research. | §4.6–§4.8 (Technology-Specific Considerations → Conclusions) |
| **OTHER INFORMATION** | | |  |
| Registration and protocol | 24a | Provide registration information for the review, including register name and registration number, or state that the review was not registered. | §2.1 Protocol and Registration — OSF DOI: [10.17605/OSF.IO/98Q3G]; registered 30 July 2025 |
|  | 24b | Indicate where the review protocol can be accessed, or state that a protocol was not prepared. | Same section — OSF link provided |
|  | 24c | Describe and explain any amendments to information provided at registration or in the protocol. | §2.1 — registered after data collection; no post-hoc changes |
| Support | 25 | Describe sources of financial or non-financial support for the review, and the role of the funders or sponsors in the review. | Funding section (end matter). |
| Competing interests | 26 | Declare any competing interests of review authors. | Conflicts of Interest section (end matter). |
| Availability of data, code and other materials | 27 | Report which of the following are publicly available and where they can be found: template data collection forms; data extracted from included studies; data used for all analyses; analytic code; any other materials used in the review. | Data Availability Statement (end matter). |

*From:*  Page MJ, McKenzie JE, Bossuyt PM, Boutron I, Hoffmann TC, Mulrow CD, et al. The PRISMA 2020 statement: an updated guideline for reporting systematic reviews. BMJ 2021;372:n71. doi: 10.1136/bmj.n71. This work is licensed under CC BY 4.0. To view a copy of this license, visit <https://creativecommons.org/licenses/by/4.0/>
