# Supplementary figures and images for: Accuracy of Navigation and Robot-Assisted Systems for Dental Implant Placement: A Systematic Review
Source: Dent J (Basel). 2025 Nov 14;13(11):537. doi: 10.3390/dj13110537 (PMC12651058; doi:10.3390/dj13110537)

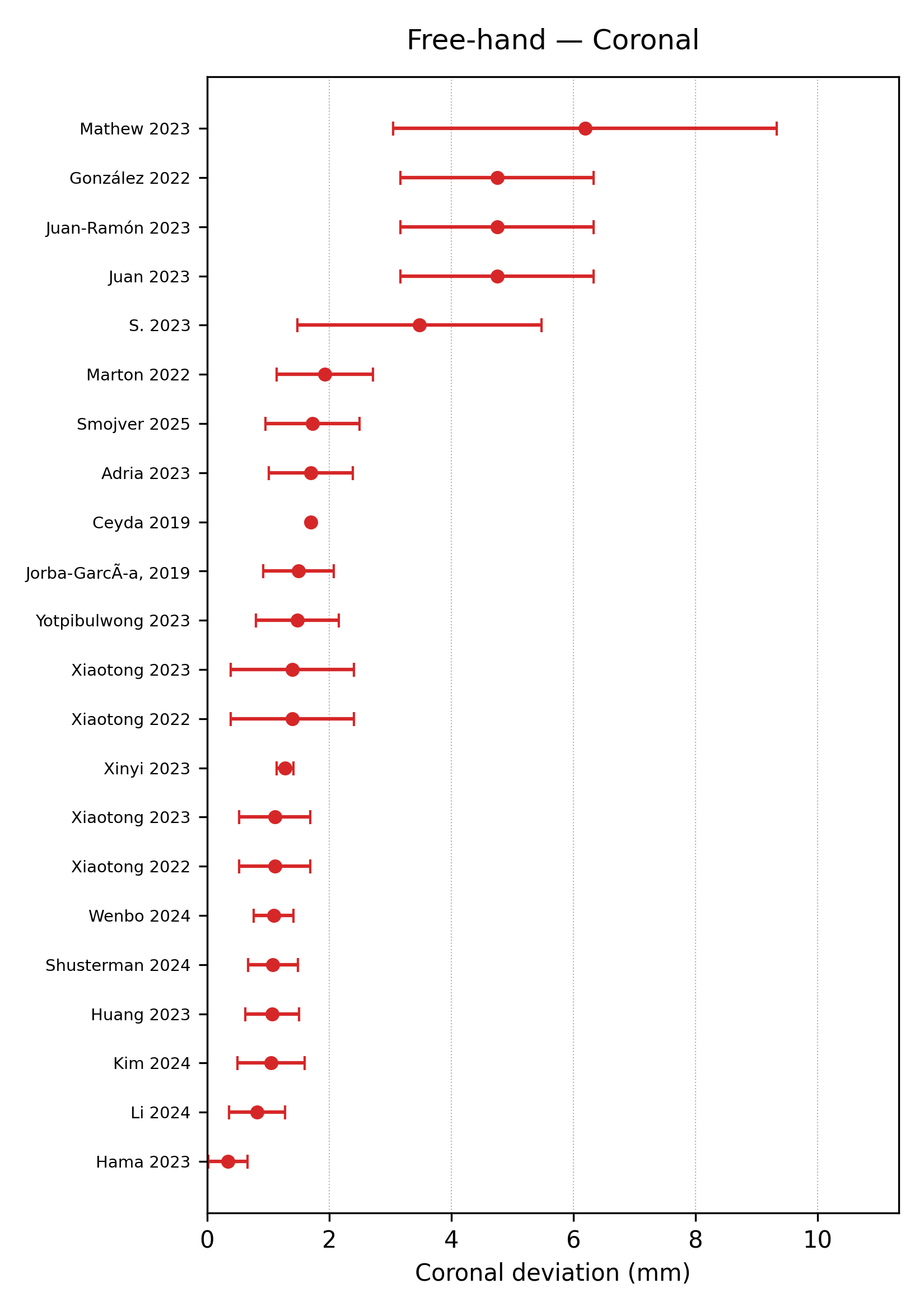

Supplement: Supplementary file 1 [file dentistry-13-00537-s001.zip › dentistry-3824564-newsupplementary/S_fig10_coronal_free-hand.png]

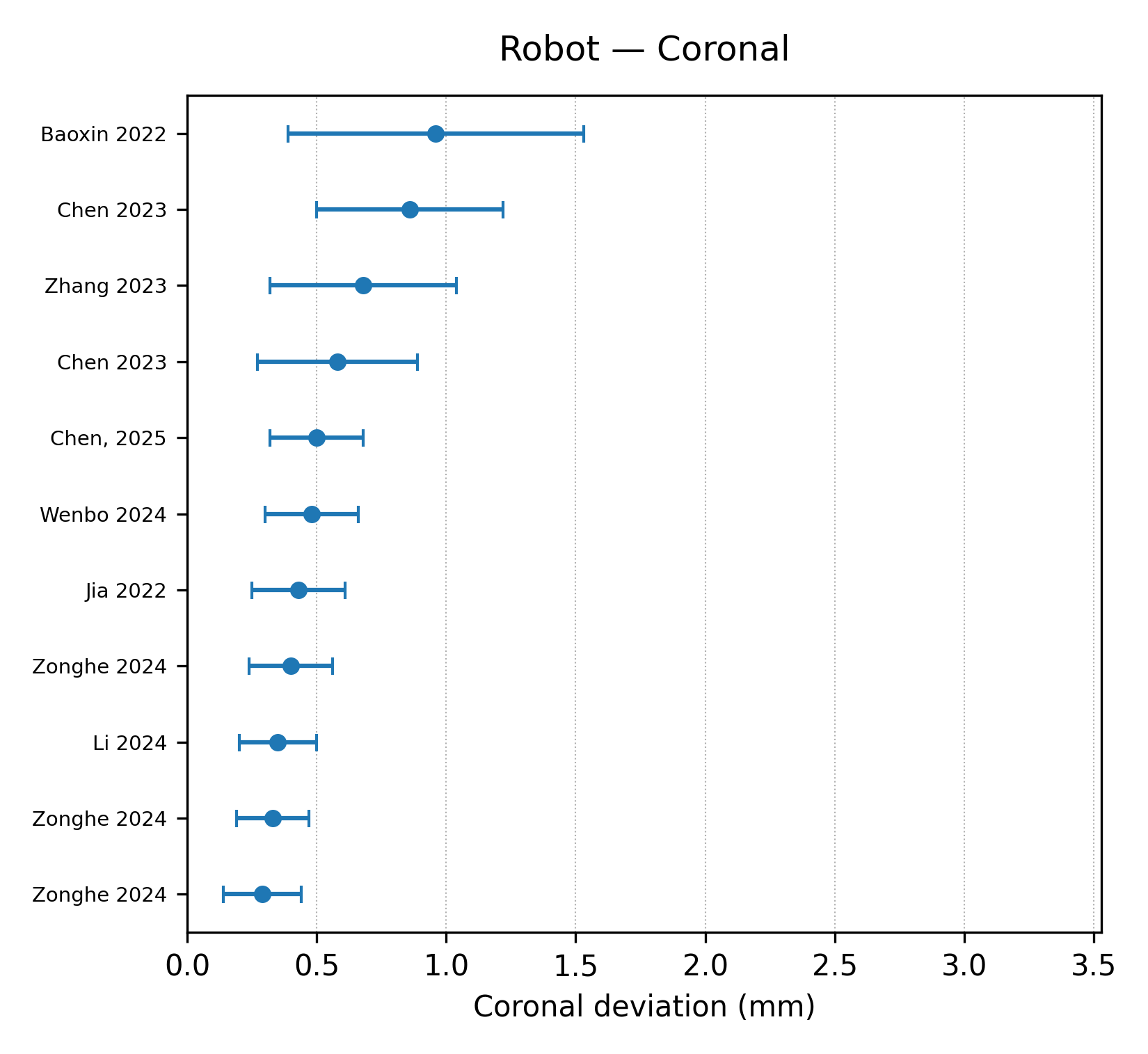

Supplement: Supplementary file 1 [file dentistry-13-00537-s001.zip › dentistry-3824564-newsupplementary/S_fig11_coronal_robot.png]

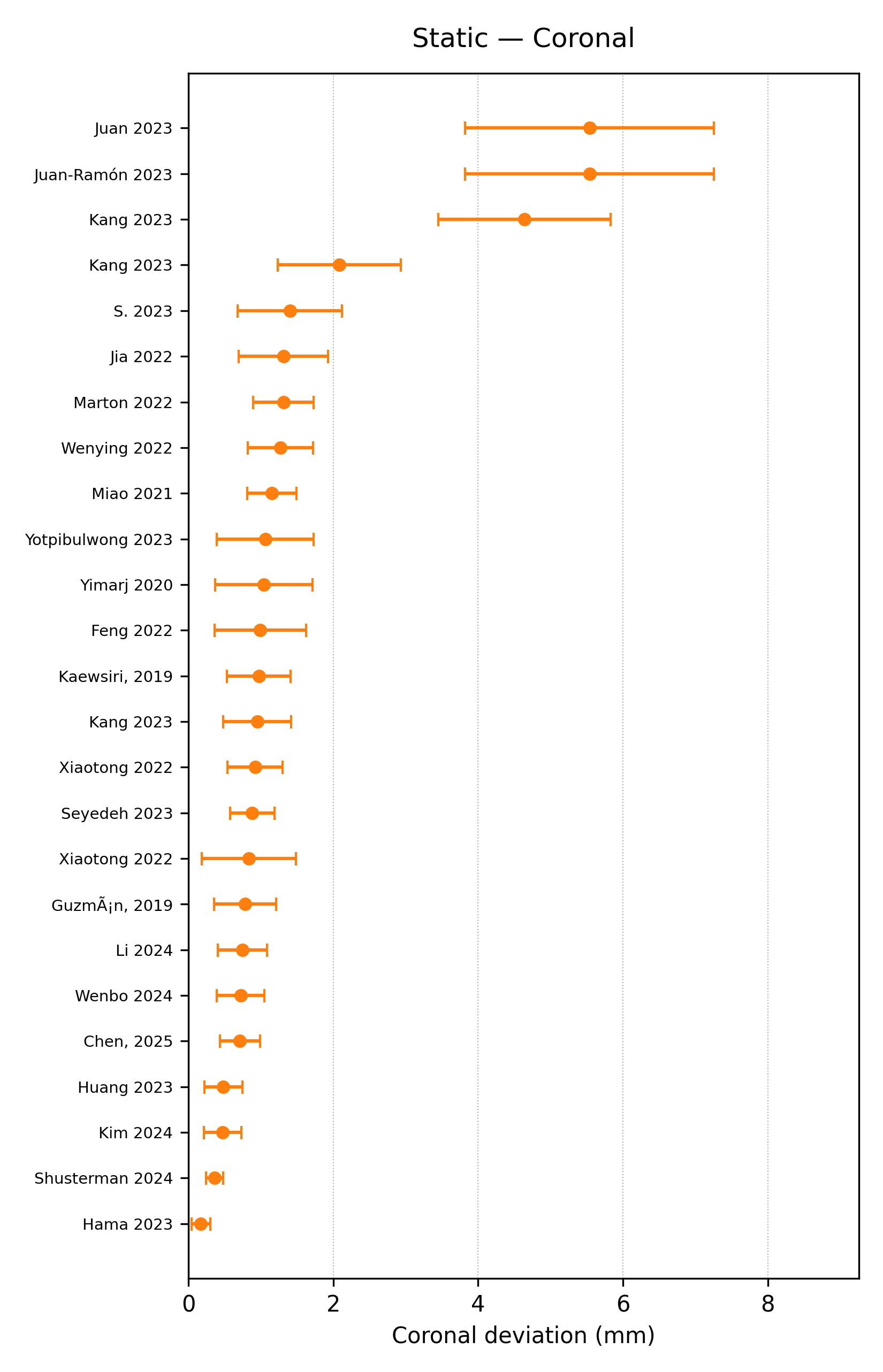

Supplement: Supplementary file 1 [file dentistry-13-00537-s001.zip › dentistry-3824564-newsupplementary/S_fig12_coronal_static.png]

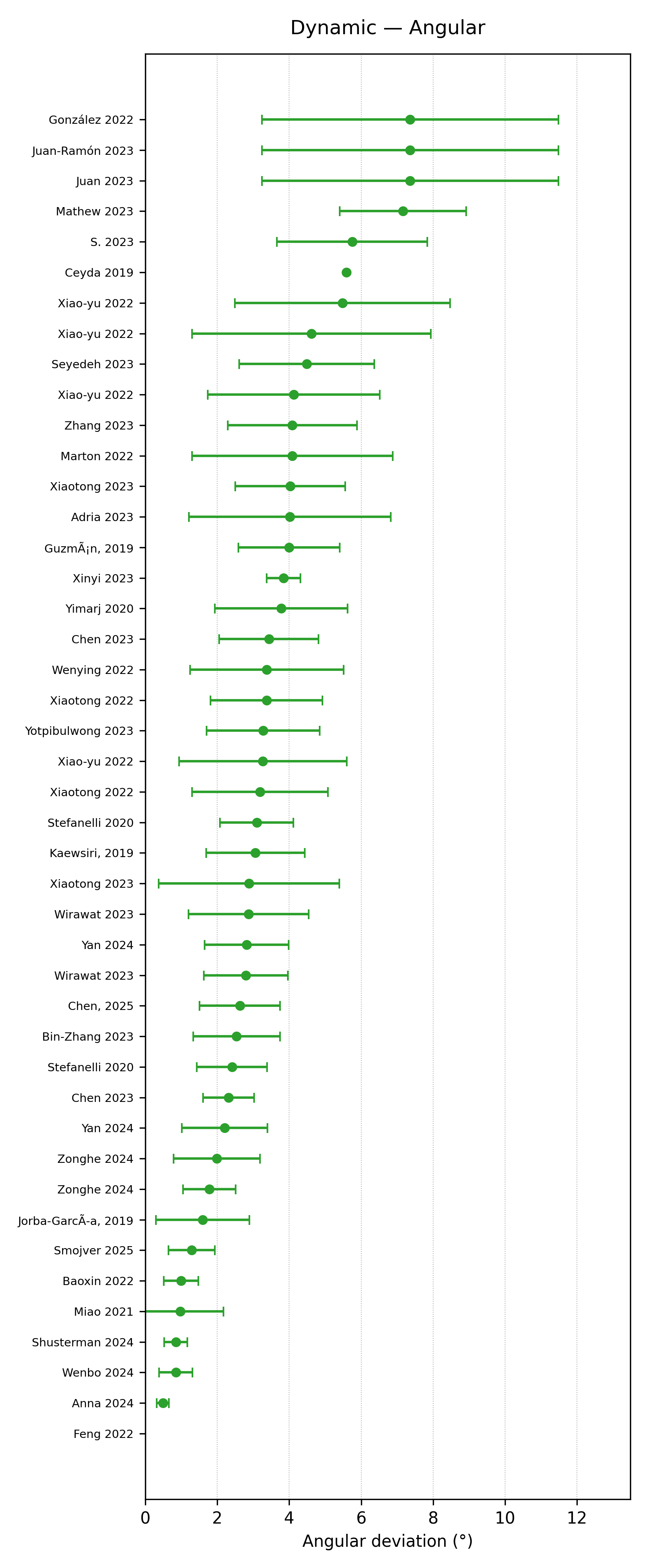

Supplement: Supplementary file 1 [file dentistry-13-00537-s001.zip › dentistry-3824564-newsupplementary/S_fig1_angular_dynamic.png]

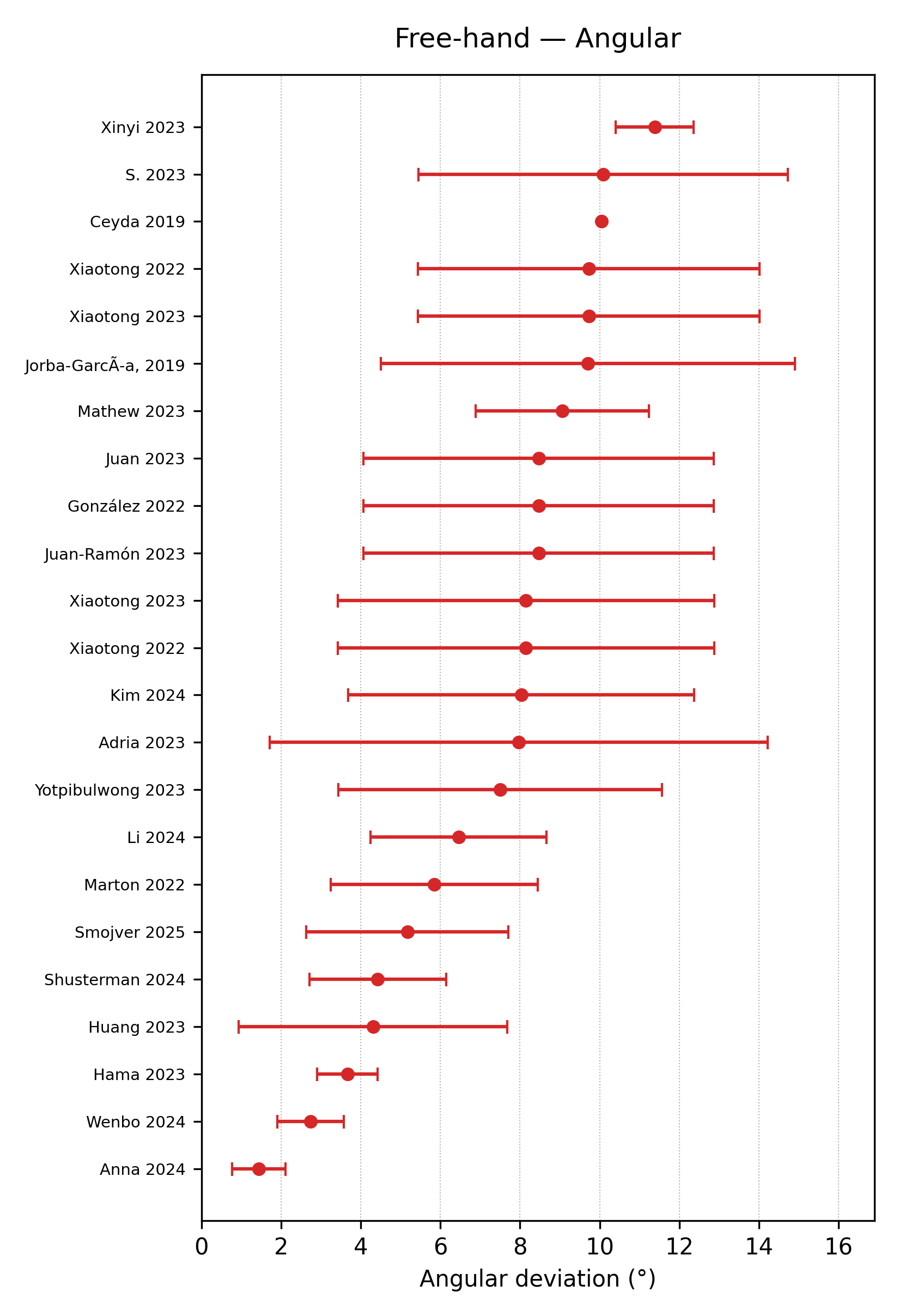

Supplement: Supplementary file 1 [file dentistry-13-00537-s001.zip › dentistry-3824564-newsupplementary/S_fig2_angular_free-hand.png]

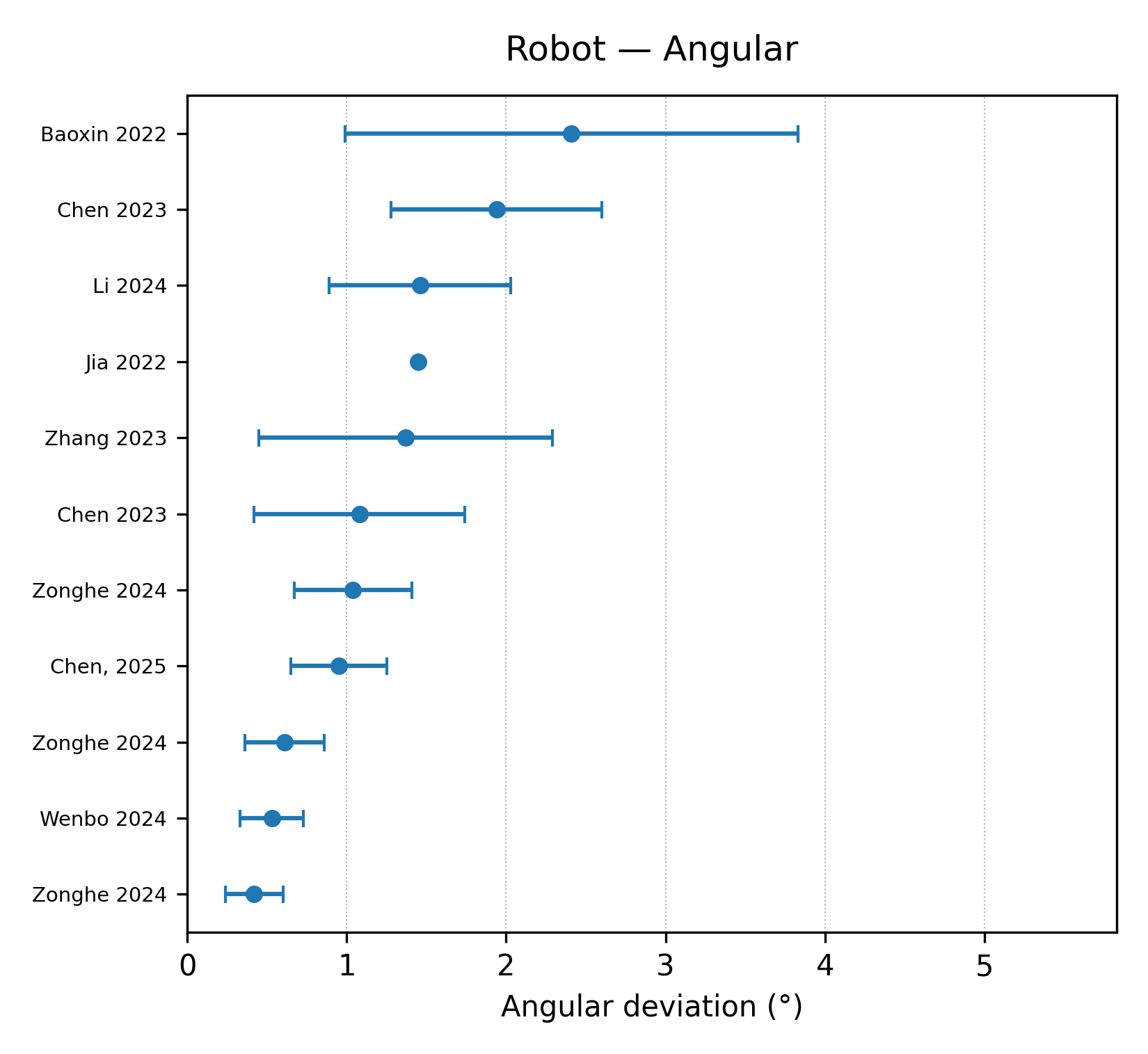

Supplement: Supplementary file 1 [file dentistry-13-00537-s001.zip › dentistry-3824564-newsupplementary/S_fig3_angular_robot.png]

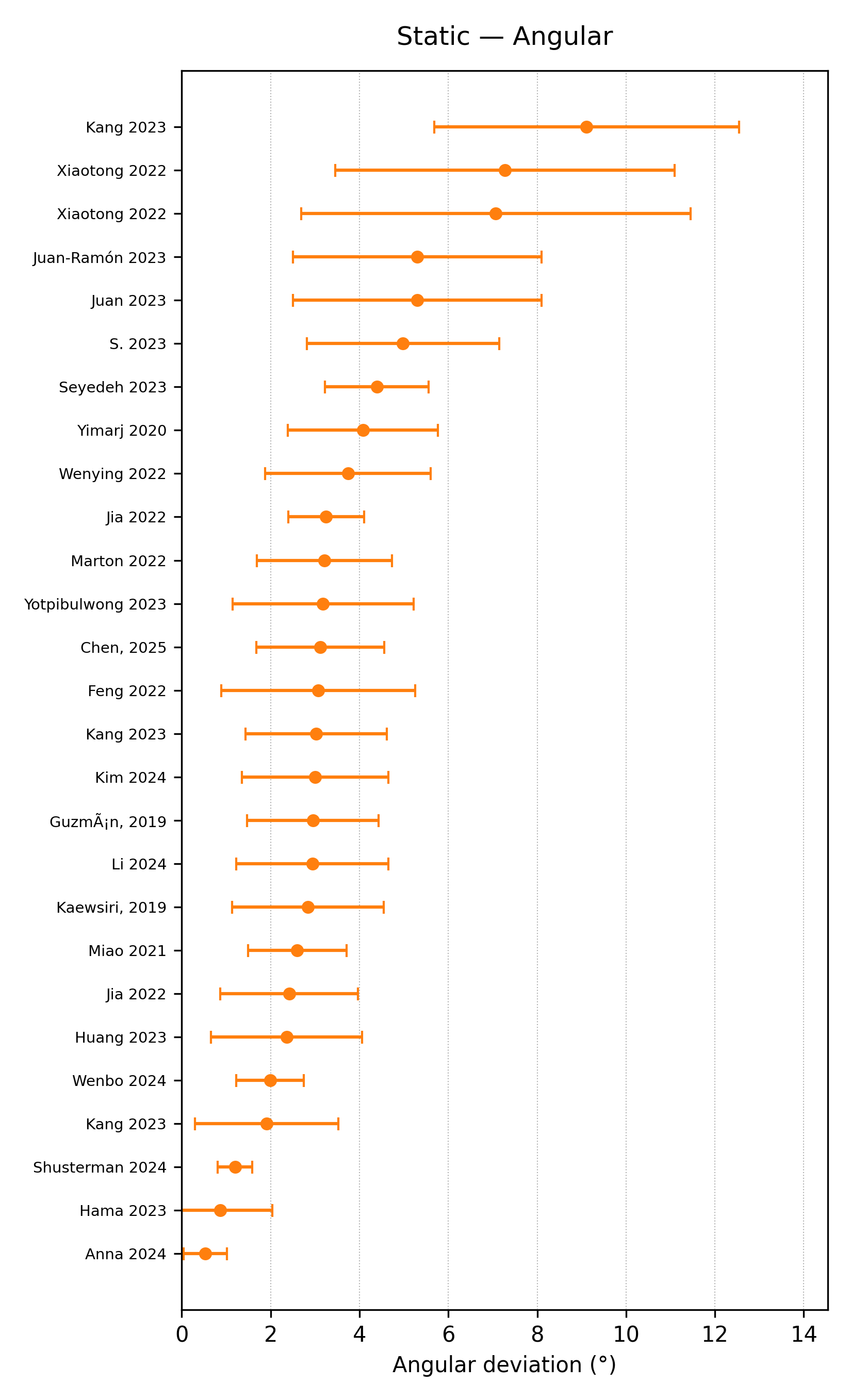

Supplement: Supplementary file 1 [file dentistry-13-00537-s001.zip › dentistry-3824564-newsupplementary/S_fig4_angular_static.png]

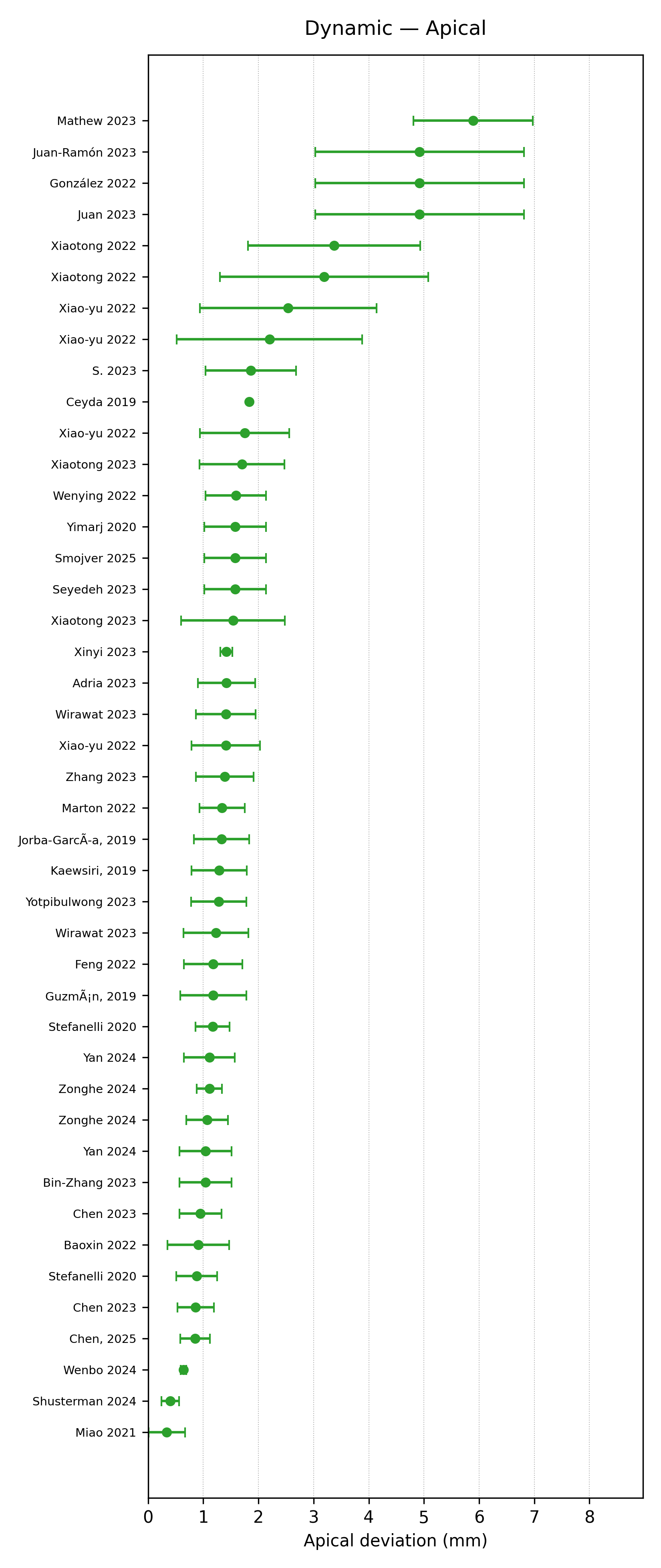

Supplement: Supplementary file 1 [file dentistry-13-00537-s001.zip › dentistry-3824564-newsupplementary/S_fig5_apical_dynamic.png]

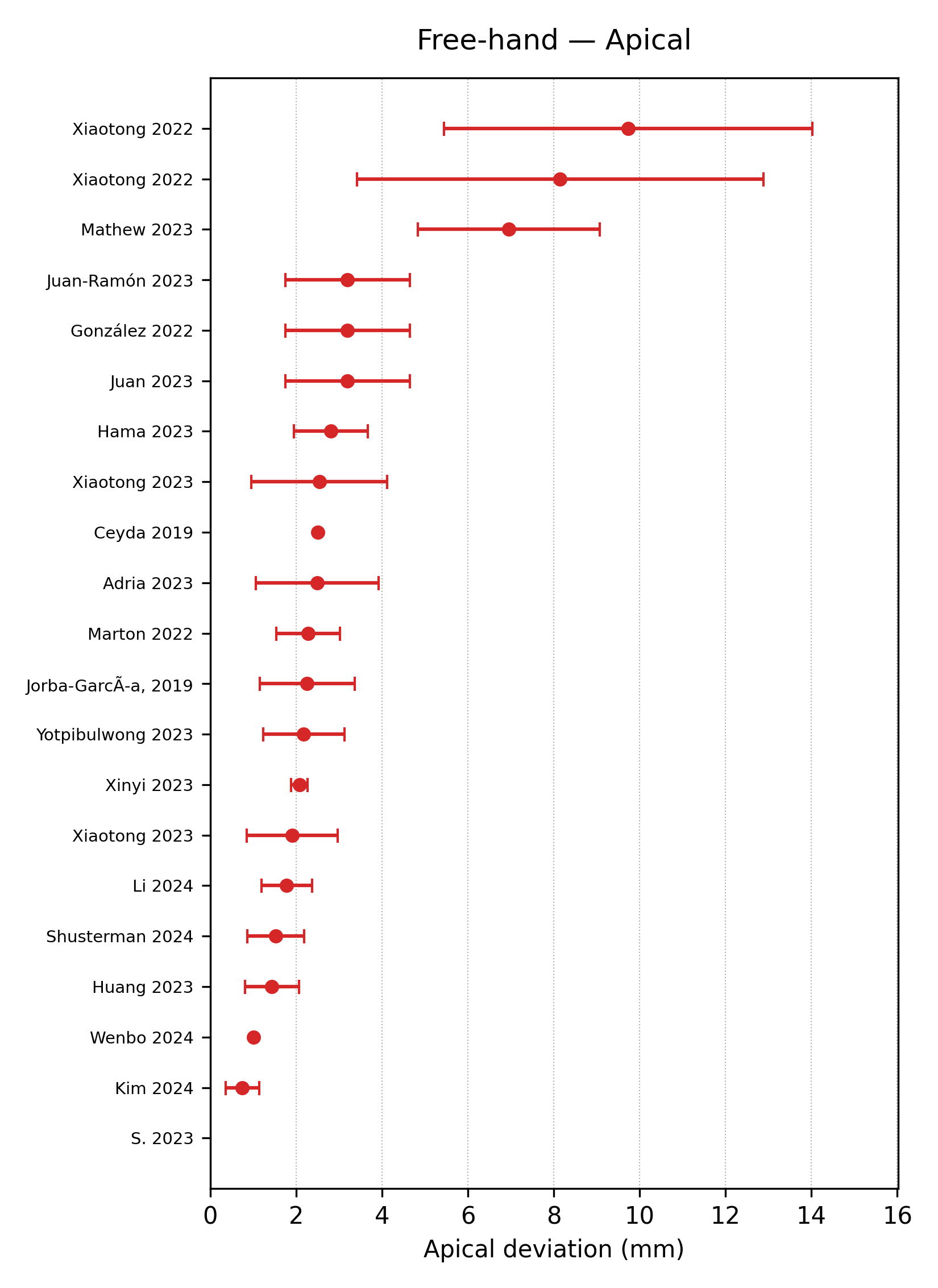

Supplement: Supplementary file 1 [file dentistry-13-00537-s001.zip › dentistry-3824564-newsupplementary/S_fig6_apical_free-hand.png]

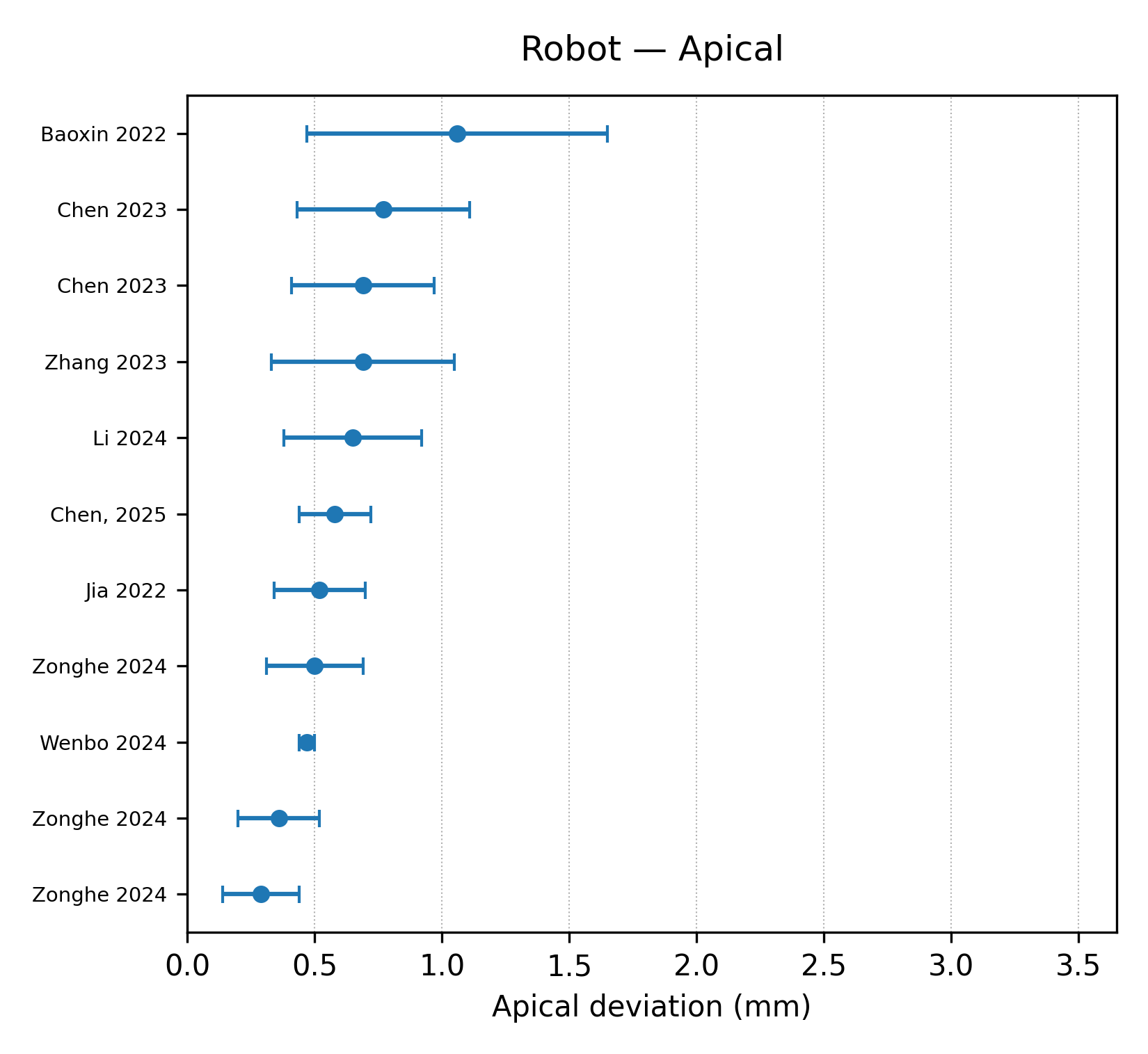

Supplement: Supplementary file 1 [file dentistry-13-00537-s001.zip › dentistry-3824564-newsupplementary/S_fig7_apical_robot.png]

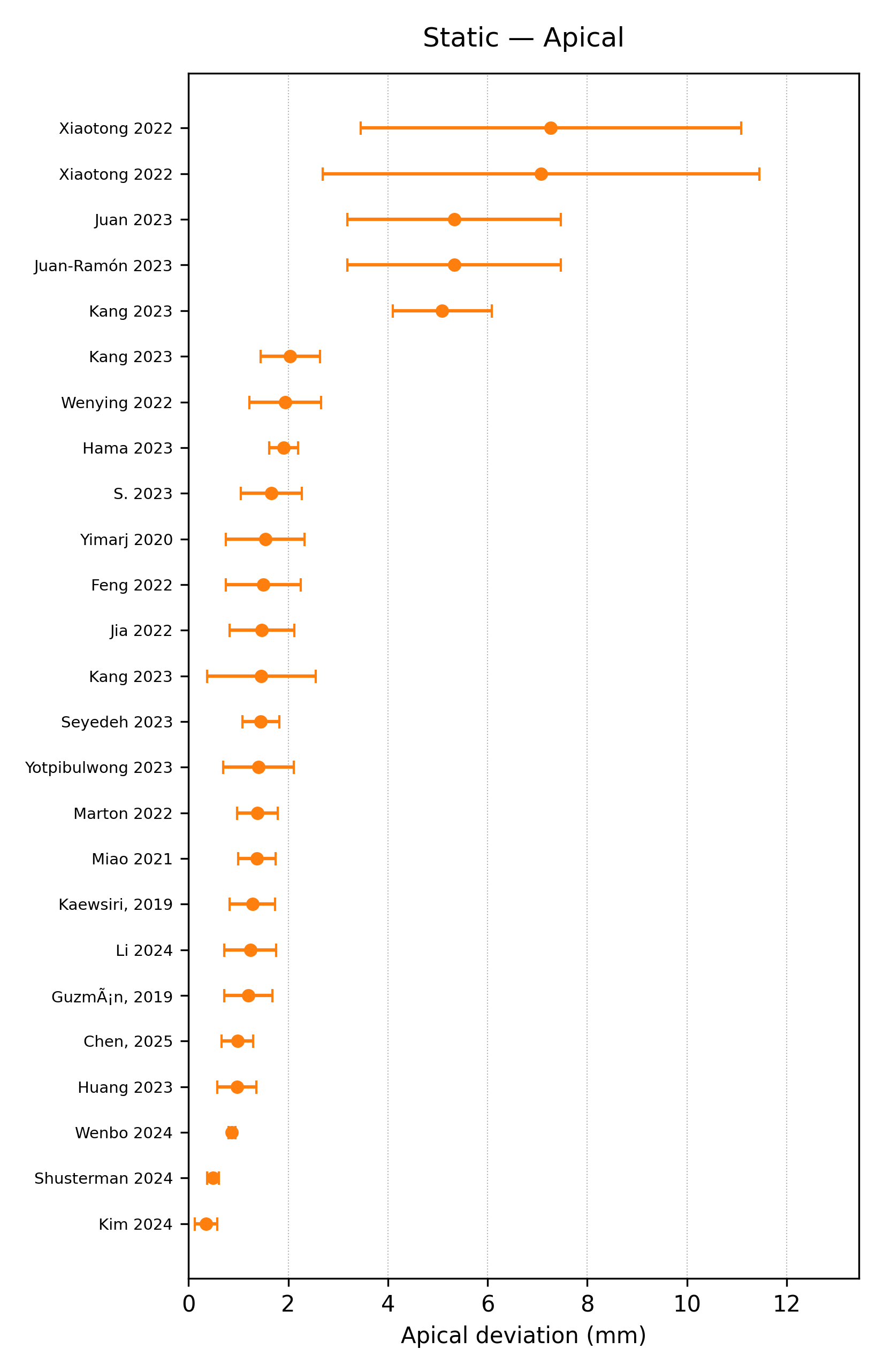

Supplement: Supplementary file 1 [file dentistry-13-00537-s001.zip › dentistry-3824564-newsupplementary/S_fig8_apical_static.png]

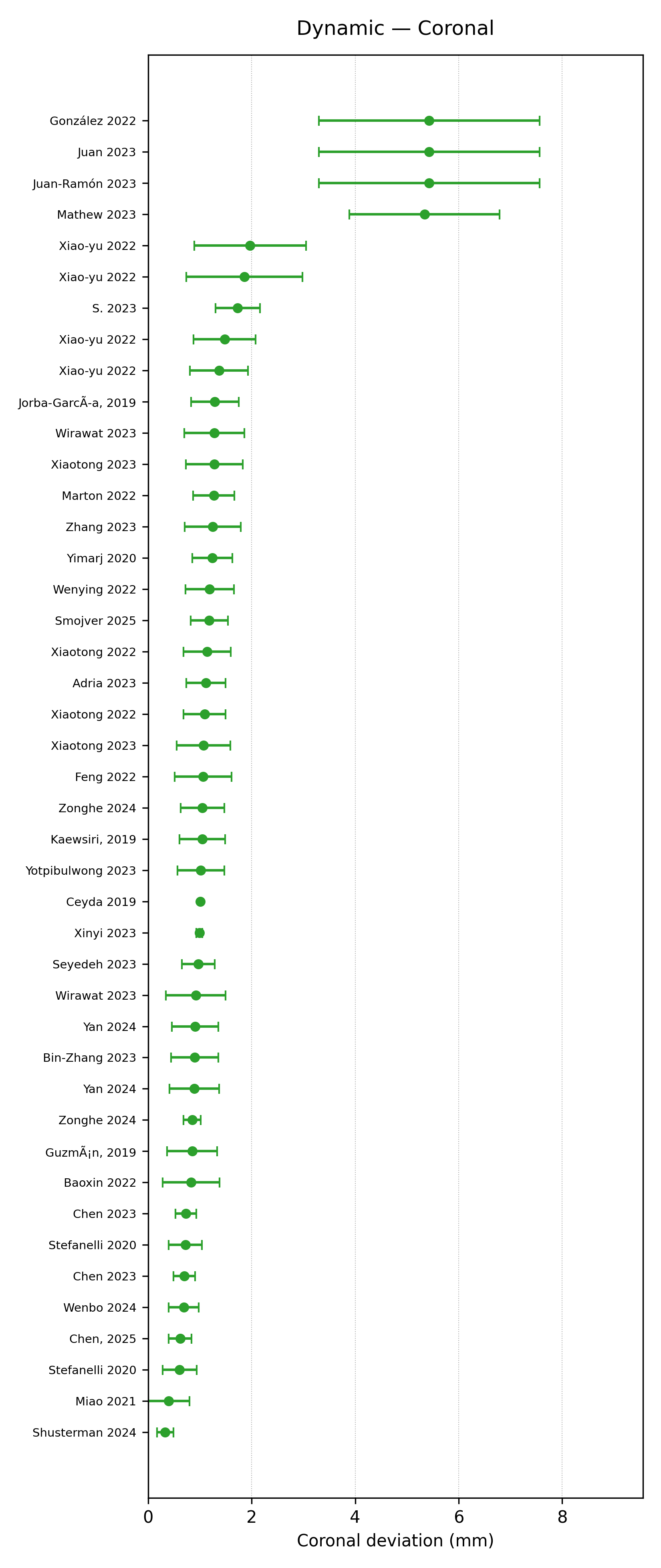

Supplement: Supplementary file 1 [file dentistry-13-00537-s001.zip › dentistry-3824564-newsupplementary/S_fig9_coronal_dynamic.png]
